# Phosphorus dendron nanomicelles as a platform for combination anti-inflammatory and antioxidative therapy of acute lung injury

**DOI:** 10.7150/thno.70701

**Published:** 2022-04-11

**Authors:** Jin Li, Liang Chen, Changsheng Li, Yu Fan, Mengsi Zhan, Huxiao Sun, Serge Mignani, Jean-Pierre Majoral, Mingwu Shen, Xiangyang Shi

**Affiliations:** 1 State Key Laboratory for Modification of Chemical Fibers and Polymer Materials, Shanghai Engineering Research Center of Nano-Biomaterials and Regenerative Medicine, College of Chemistry, Chemical Engineering and Biotechnology, Donghua University, Shanghai 201620, People's Republic of China; 2 Laboratoire de Chimie de Coordination du CNRS, 205 Route de Narbonne, BP 44099, 31077 Toulouse CEDEX 4, France; 3 Université de Toulouse, UPS, INPT, 31077 Toulouse CEDEX 4, France; 4 Université Paris Descartes, PRES Sorbonne Paris Cité, CNRS UMR 860, Laboratoire de Chimie et de Biochimie Pharmacologiques et Toxicologique, 45, rue des Saints Pères, 75006 Paris, France; 5 CQM-Centro de Quimica da Madeira, Universidade da Madeira, 9020-105 Funchal, Portugal

**Keywords:** phosphorus dendrons, nanomicelles, curcumin, alveolar macrophages, NF-κB, acute lung injury

## Abstract

**Rationale:** Development of novel nanomedicines to inhibit pro-inflammatory cytokine expression and reactive oxygen species (ROS) generation for anti-inflammatory therapy of acute lung injury (ALI) remains challenging. Here, we present a new nanomedicine platform based on tyramine-bearing two dimethylphosphonate sodium salt (TBP)-modified amphiphilic phosphorus dendron (C11G3) nanomicelles encapsulated with antioxidant drug curcumin (Cur).

**Methods:** C11G3-TBP dendrons were synthesized *via* divergent synthesis and self-assembled to generate nanomicelles in a water environment to load hydrophobic drug Cur. The created C11G3-TBP@Cur nanomicelles were well characterized and systematically examined in their cytotoxicity, cellular uptake, intracellular ROS elimination, pro-inflammatory cytokine inhibition and alveolar macrophages M2 type repolarization *in vitro*, and evaluated to assay their anti-inflammatory and antioxidative therapy effects of ALI mice model through pro-inflammatory cytokine expression level in bronchoalveolar lavage fluid and lung tissue, histological analysis and micro-CT imaging detection of lung tissue injury *in vivo*.

**Results:** The nanomicelles with rigid phosphorous dendron structure enable high-capacity and stable Cur loading. Very strikingly, the drug-free C11G3-TBP micelles exhibit excellent cytocompatibility and intrinsic anti-inflammatory activity through inhibition of nuclear transcription factor-*kappa* B, thus causing repolarization of alveolar macrophages from M1 type to anti-inflammatory M2 type. Taken together with the strong ROS scavenging property of the encapsulated Cur, the developed nanomicelles enable effective therapy of inflammatory alveolar macrophages *in vitro* and an ALI mouse model *in vivo* after atomization administration.

**Conclusion:** The created phosphorus dendron nanomicelles can be developed as a general nanomedicine platform for combination anti-inflammatory and antioxidative therapy of inflammatory diseases.

## Introduction

Improved understanding of pathogenesis in acute lung injury (ALI) has led to discovery of novel therapeutic targets such as pro-inflammatory cytokines 1, 2, including tumor necrosis factor-α (TNF-α), interleukin-1β (IL-1β), and interleukin-6 (IL-6), and reactive oxygen species (ROS) 3, which are all secreted by M1-type alveolar macrophages. The overexpressed pro-inflammatory cytokines of M1 type alveolar macrophages are able to promote an inflammatory cascade that causes neutrophils to migrate continuously to the lungs for inflammatory infiltration 4. Meanwhile, the neutrophils could directly induce epithelial and endothelial cell death, resulting in lung damage and pneumoneadema 5, 6. In addition, overexpressed ROS in the macrophages has been regarded to mediate inflammation to modulate vascular endothelial cells, leading to increased pulmonary vascular permeability 7. Therefore, it is of paramount significance to create effective formulations to down-regulate both pro-inflammatory cytokines and ROS. As opposed to M1-type alveolar macrophages, M2-type alveolar macrophages are known to secrete anti-inflammatory cytokines such as cluster of differentiation 206 (CD206), arginase-1 (Arg-1), and interleukin-10 (IL-10), and to phagocytose neutrophils to repair damaged lung tissues 2, 8. Therefore, it has been of great importance to develop new formulations that can repolarize M1-type macrophages to M2-type ones for effective ALI therapy.

Practically, anti-inflammatory drugs, such as glucocorticoid 9, 10, antitussive expectorant 11, antibiotics 12, and immunosuppressor 13, have been widely investigated for ALI therapy. For instance, antioxidative drugs such as curcumin (Cur) 14, 15, resveratrol 16 and vitamin E 17 have been employed as ROS scavenger drugs. Unfortunately, these drugs suffer problems of water-insolubility, low bioavailability and undesired side effects, quite limiting their applications in ALI therapy. In particular, as an active ingredient of traditional Chinese medicine, Cur can effectively inhibit the secretion of pro-inflammatory cytokines and reduce ROS level of M1-type alveolar macrophages 18 due to its two hydroxyl groups on the phenylacrylyl skeleton that can interact with oxygen radicals to form stable quinone radicals 19, 20. In order to overcome the common limitations in the clinical applications of Cur due to its poor water solubility and low bioavailability, it is urgent to develop versatile Cur-loaded nanomedicine formulations for effective ALI therapy.

Nanocarriers have distinctive advantages in solving the defects of conventional chemotherapy of ALI to improve the water solubility and bioavailability of drugs. A range of nanoplatforms including liposomes 21, chitosan nanoparticles (NPs) 22, microspheres 15, dendrimers 23, 24, and nanomicelles 16 have been developed for ALI chemotherapy. For instance, polyethylene glycol-modified poly(lactic-co-glycolic acid) was used to fabricate Cur-loaded microspheres with excellent biocompatibility and biodegradability to enhance the Cur bioavailability for improved ALI chemotherapy 15. On the other hand, some nanomaterials such as inorganic CeO_2_ or Se/SiO_2_ NPs 25, 26, polydopamine NPs 27 and phosphorus dendrimers 28 display inherent anti-inflammatory or antioxidative activity that have been proposed for ALI treatment. Particularly, phosphorus dendrimers with well-defined architecture and molecular geometry have been emerging as a promising candidate used for ALI therapy. For example, generation 3 (G3) poly(phosphorhydrazone) dendrimers grafted with mannose have been proven to bind lipopolysaccharide (LPS)-activated dendritic cells (DCs) in lung injury lesions, and prevent lung inflammation by reducing alveolar wall thickening and neutrophil influx through inhibition of the pro-inflammatory cytokine TNF-α in DCs 28. In another work 29, bisphosphonate-modified G3 and G4 phosphorus dendrimers have been proven to repolarize peritoneal macrophages to M2 type through inhibition of nuclear transcription factor-*kappa* B (NF-κB) and downregulation of inflammatory mediator nitric oxide (NO) for excellent anti-inflammatory therapy of air pouch mice models. Noticeably, the developed phosphorus dendrimers lack water solubility to limit their further applications, and meanwhile, there are no reports in the literature dealing with the use of phosphorus dendrimers to combine with drugs for efficient combination therapy of ALI.

For effective treatment of ALI, it is desirable to develop combination therapy formulations to tackle intricate biological pathways in one single shot. Recently 30, we presented a folic acid-targeted multifunctional G5 poly(amidoamine) dendrimer-based platform to co-deliver anti-inflammatory TNF-α siRNA and antioxidative alpha-tocopheryl succinate to macrophage cells for targeted combination therapy of rheumatoid arthritis. This study revealed a judicious strategy of combination anti-inflammatory and antioxidative therapy of dysfunctional macrophages triggering inflammatory diseases. It is reasonable to hypothesize that nanoplatforms with inherent anti-inflammatory activity can be developed to load chemical drugs to achieve not only repolarization of alveolar macrophages to M2 type, but also efficient elimination of ROS in the alveolar macrophages for effective ALI therapy.

**Figure 1:**
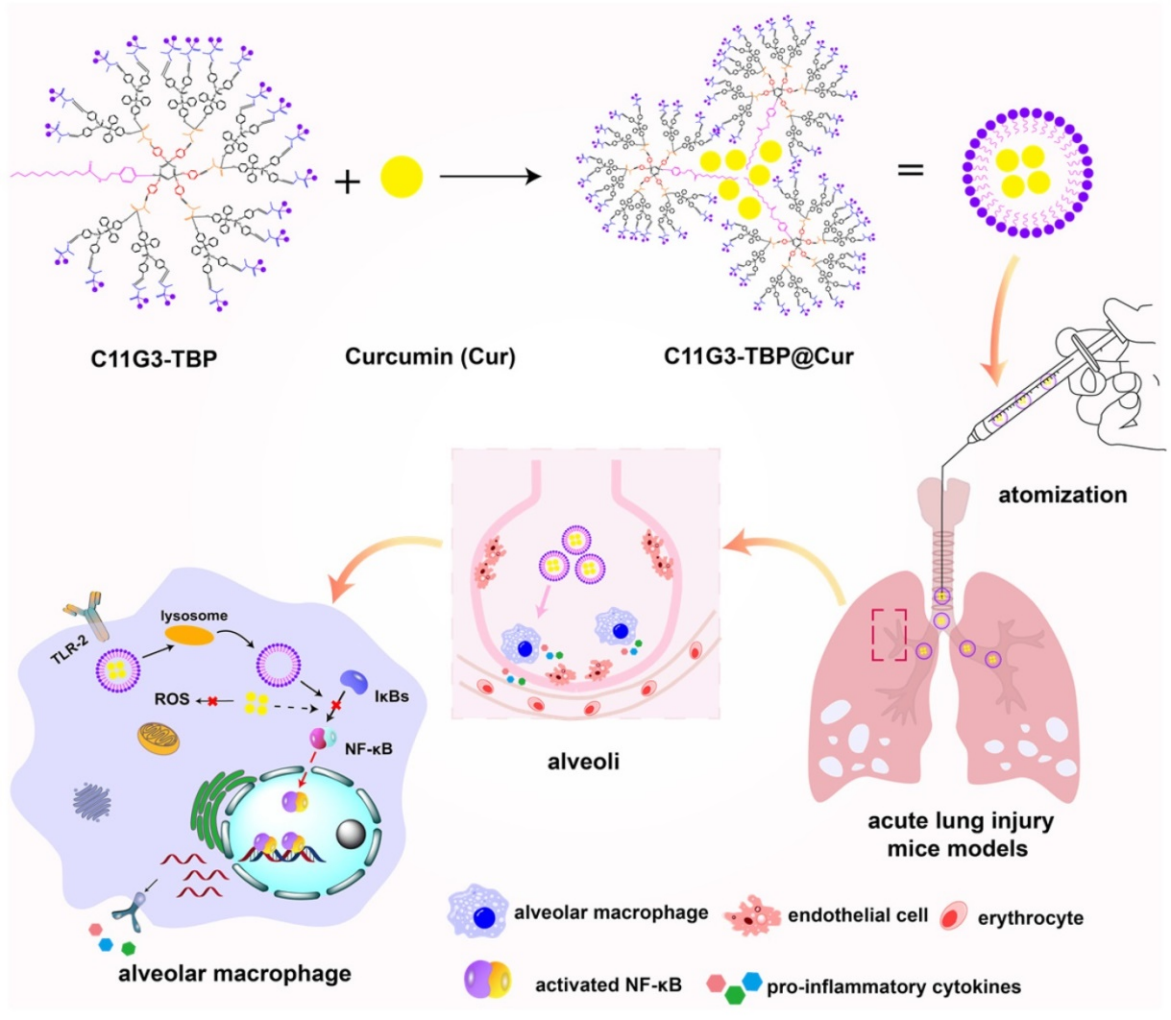
Schematic illustration of the C11G3-TBP@Cur nanomicelles for combined anti-inflammatory and antioxidative ALI therapy. C11G3-TBP nanomicelles formed* via* self-assembly method were encapsulated with Cur to obtain C11G3-TBP@Cur nanomicelles for the treatment of pro-inflammatory alveolar macrophage-induced ALI.

In the current investigation, we attempted to develop G3 amphiphilic phosphorus dendron-based nanomicelles with intrinsic anti-inflammatory activity as a platform to load Cur for enhanced ALI combination therapy (Figure 1). G3 phosphorus dendrons with hydrophobic alkyl chain (C_11_H_23_) as cores were first synthesized through a divergent method, surface modified with bisphosphonate groups, and then hydrolyzed to obtain tyramine-bearing two dimethylphosphonate sodium salt (TBP)-terminated anionic amphiphilic phosphorus dendrons (C11G3-TBP, Figure S1). The thus generated C11G3-TBP dendrons were self-assembled to generate nanomicelles in a water environment (Figure S2), and used as a vector to load hydrophobic drug Cur. The formation of dendrons, nanomicelles, and drug-loaded nanomicelles were thoroughly characterized. Lastly, drug-loaded nanomicelles were used to treat LPS-activated alveolar macrophages *in vitro* and the LPS-induced ALI mouse model *in vivo*. According to our knowledge, our study represents the very first demonstration to adopt the inherent anti-inflammatory amphiphilic phosphorus dendron-based nanomicelles for combined anti-inflammatory and antioxidative ALI therapy.

## Results and Discussion

### Preparation and characterization of amphiphilic phosphorus dendron nanomicelles for Cur encapsulation

**Figure 2:**
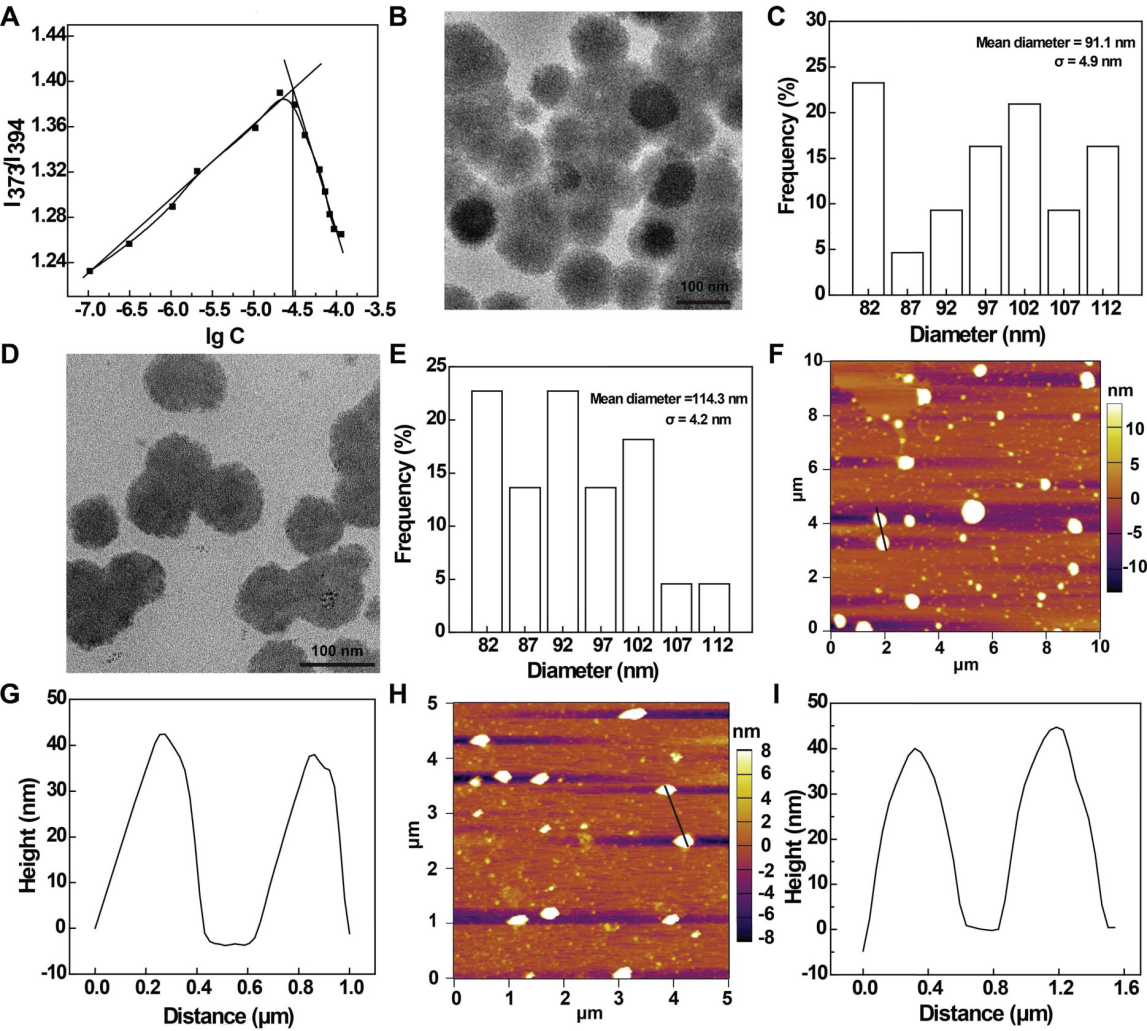
(A) Determination of the CMC of anionic phosphorous dendrons of C11G3-TBP using a fluorescent dye pyrene. TEM image and size distribution histogram of (B-C) C11G3-TBP and (D-E) C11G3-TBP@Cur micelles. AFM image and corresponding height profile of (F-G) C11G3-TBP and (H-I) C11G3-TBP@Cur micelles.

In this work, we prepared a novel amphiphilic phosphorus dendrons with hydrophobic alkyl chain (C_11_H_23_), rigid structure and dimethylphosphonate hydrophilic group according to the literature 31, 32. The detailed synthesis steps are shown in Figure S1. The created anionic dendrons were thoroughly characterized *via* different NMR techniques (Figure S3-S10, see details in the Supplementary Materials). With the amphiphilic nature quite similar to phosphorous dendrons described in our previous report 33, we expect that dendron micelles can be formed through self-assembly. Hence, the critical micelle concentration (CMC) of the C11G3-TBP dendrons was measured using pyrene as a fluorescent probe (Figure 2A), and the CMC was measured to be 27.35 μM. The formation of C11G3-TBP dendron micelles was revealed by transmission electron microscopy (TEM) imaging and dynamic light-scattering (DLS). As can be observed from a representative TEM image, the micelles show a spheroidal morphology with a mean diameter of 91.1 nm (Figure 2B-C). DLS and zeta potential measurements show that the C11G3-TBP micelles have a hydrodynamic size around 122.2 nm (Figure S11A) and a negatively charged surface potential of -42.5 ± 3.3 mV (Figure S11B).

Next, we used the C11G3-TBP nanomicelles as a carrier to load an antioxidative drug Cur through self-assembly. The drug loading content (LC) and encapsulation efficiency (EE) of C11G3-TBP@Cur nanomicelles were investigated by varying the C11G3-TBP/Cur molar ratio. As indicated in Figure S12A, the optimal LC and EE can be attained up to 21.24% and 96.86% at the optimal molar ratio of 20: 1. This high LC and EE could be owing to the unique rigid molecular phosphorous dendritic backbone structure and sufficient hydrophobic internal cavity of micelles. The Cur release from the C11G3-TBP@Cur nanomicelles was also investigated. As depicted in Figure S12B**,** C11G3-TBP@Cur micelles can slowly release Cur in a time-dependent manner and 20% of total Cur can be released in 9 days. Since the initial burst release is only about 5.8% within the first day, the C11G3-TBP@Cur micelles could effectively prevent Cur leakage for efficient intracellular lysosome uptake to exert its therapeutic function 34. Furthermore, the morphology and size of the C11G3-TBP@Cur micelles were measured by TEM and DLS. The resulting C11G3-TBP@Cur micelles display the same morphology as the drug-free micelles, but with increased mean diameter of 114.3 nm (Figure 2D-E) and hydrodynamic size of 199.2 nm (Figure S11A), which should be contributed by the drug loading to expand the micellar spatial structure 35.

Meanwhile, it should be noted that the nanomicelles can maintain their nanoscale dimension even after they were diluted at a concentration much lower than their CMC. As indicated by DLS analysis (Figure S11A), the C11G3-TBP@Cur nanomicelles at a concentration of 4.17 μM (much lower than their CMC) display a hydrodynamic size of 213.5 nm, slightly larger than those at a concentration of 41.7 μM. This could be due to the fact that at a diluted state, the micelles tend to be more expanded than those in a squeezed state in the same space. In any case, the more or less similar hydrodynamic size of the C11G3-TBP@Cur nanomicelles even in a diluted state means that the drug-loaded nanomicelles have a good structural stability, which can also be supported by their consistent surface potentials (Figure S11B). Both C11G3-TBP and C11G3-TBP@Cur micelles display good colloidal stability after they were exposed to different aqueous media (water, phosphate buffered saline (PBS) or cell culture medium) at 4 ℃ for at least one week (Figure S13A-B). We also used the DLS assay to check their hydrodynamic size changes in water. Both C11G3-TBP and C11G3-TBP@Cur do not display obvious hydrodynamic size changes for at least 7 days (Figure S13C). Furthermore, atomic force microscope (AFM) imaging was carried out to check the micellar morphology change after Cur encapsulation (Figure 2F and Figure 2H). It is evident that the Cur loading does not lead to any appreciable micellar structure changes, and the heights of the C11G3-TBP (39.1 nm) and C11G3-TBP@Cur (40.2 nm) micelles are quite consistent (Figure 2G and Figure 2I).

### Anti-inflammatory therapy of alveolar macrophages *in vitro*

**Figure 3:**
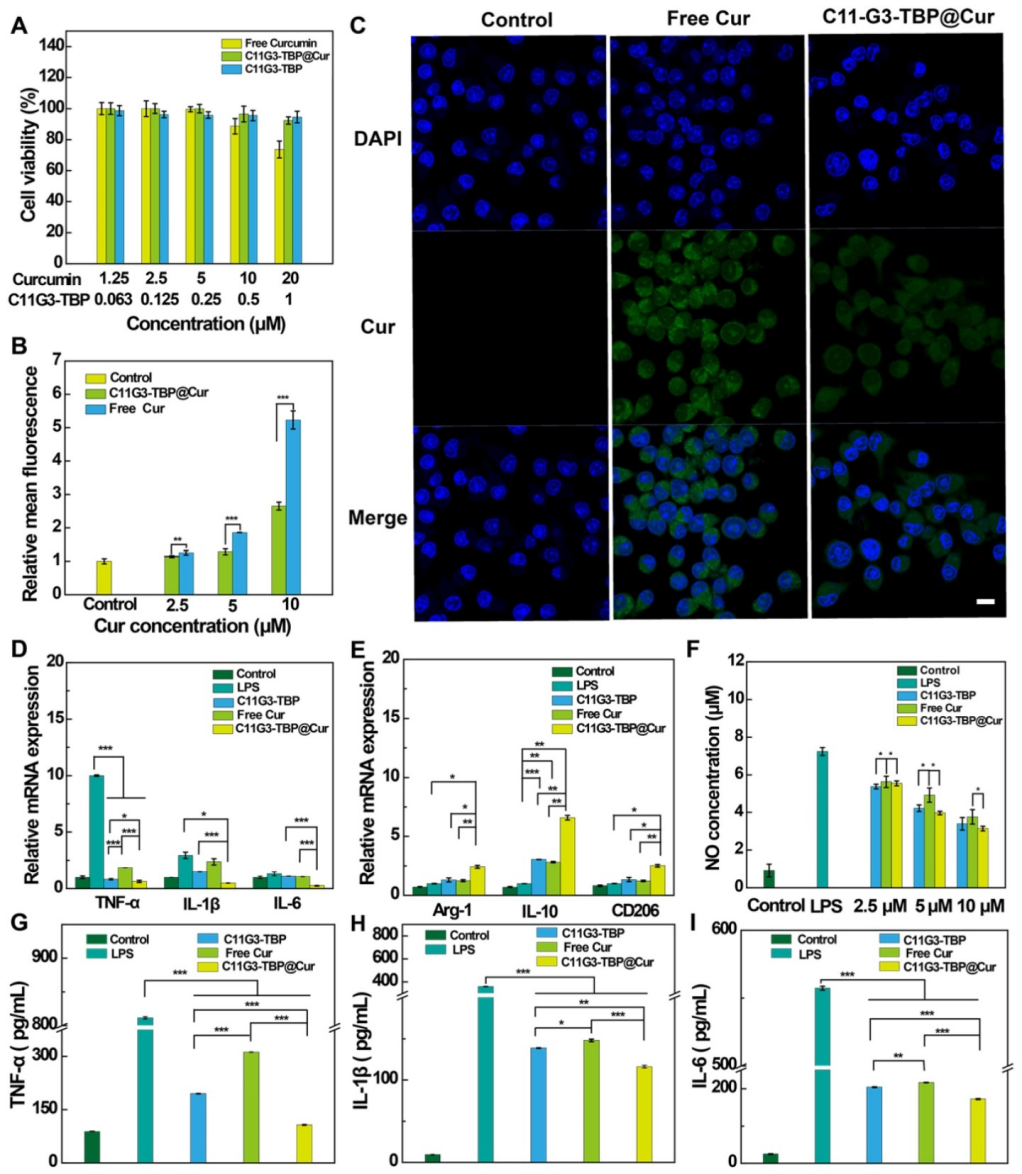
(A) CCK-8 viability assay of activated MH-S cells treated with C11G3-TBP, C11G3-TBP@Cur and free Cur (n = 5). (B) Quantitative flow cytometry assay of activated MH-S cells incubated with C11G3-TBP@Cur and free Cur, respectively at different Cur concentrations for 4 h. The fluorescence intensity of control group was set to be 1 (n = 3). (C) Confocal microscopic imaging of activated MH-S cells treated with C11G3-TBP@Cur ([Cur] = 10 μΜ) and free Cur (10 μΜ) for 4 h. The scale bar in each panel represents 10 μm. RT-PCR assay of (D) M1-type macrophage marker (TNF-α, IL-1β and IL-6) and (E) M2-type macrophage marker (Arg-1, IL-10 and CD206) expression. (F) Inflammation mediator (NO) level in cell culture medium after the cells were treated with different materials at different equivalent Cur concentrations. ELISA assay of the expression of pro-inflammatory cytokines of (G) TNF-α, (H) IL-1β, and (I) IL-6 in cell medium, respectively. Normal MH-S cells and LPS-activated MH-S cells both treated with PBS were used as negative and positive controls, respectively.

Before we tested the bioactivity of the anionic nanomicelles, the cytotoxicity of the C11G3-TBP and C11G3-TBP@Cur nanomicelles was examined by CCK-8 viability assay of activated mouse alveolar macrophage (MH-S) cells (Figure 3A). Obviously, as opposed to the treatment of free Cur that can cause decreased cell viability to 73.6% at the Cur concentration of 20 μM, cells treated with both C11G3-TBP and C11G3-TBP@Cur do not seem to have more significantly reduced viability at the equivalent Cur concentrations than the PBS control. This suggests that both drug-free and drug-loaded micelles possess excellent cytocompatibility at the studied concentrations.

For successful delivery of Cur to macrophage cells, we firstly checked the cellular uptake of C11G3-TBP@Cur or free Cur through flow cytometry (Figure 3B). Cells treated with either C11G3-TBP@Cur or free Cur display dose-dependent Cur fluorescence intensity, indicating their efficient uptake of Cur. Since the fluorescence intensity of free Cur is about 2.1 times higher than that of C11G3-TBP@Cur at an equivalent Cur concentration due to the quenching effect (Figure S14), cells treated with the C11G3-TBP@Cur should have a higher actual Cur uptake than those treated with free Cur group especially at lower Cur concentrations of 2.5 and 5.0 μM. Furthermore, the intracellular uptake was confirmed by confocal microscopic imaging of the Cur-associated green fluorescent signals in cells (Figure 3C). In contrast to the PBS control group that only shows the 4',6-diamidino-2-phenylindole (DAPI)-stained blue fluorescent cell nuclei, cells incubated with C11G3-TBP@Cur and free Cur show the obvious green fluorescence signals according to fluorescence intensity analysis (Figure S15), in good consistence with the quantitative flow cytometry assay data.

Anti-inflammatory drugs are known to treat dysfunctional macrophages through inhibition of pro-inflammatory cytokines 36, 37. Firstly, we examined the pro-inflammatory cytokines (TNF-α, IL-1β and IL-6) in LPS-activated MH-S cells after treatment with drug-free C11G3-TBP micelles at varying concentrations (0.125, 0.25, 0.5, and 1 μM, respectively) *via* real-time polymerase chain reaction (RT-PCR). As opposed to the LPS-treated positive control group that has high expressions of three pro-inflammatory cytokines, the C11G3-TBP-treated group displays the strongest anti-inflammatory effect at a concentration of 0.5 μM to inhibit the expression of those cytokines (Figure S16).

We next examined the pro-inflammatory cytokine (M1-type macrophage marker) levels of activated MH-S cells treated with C11G3-TBP@Cur, C11G3-TBP or free Cur *via* RT-PCR (Figure 3D) and enzyme-linked immunosorbent assay (ELISA, Figure 3G-I). Apparently, in contrast to the positive control of LPS-treated cells, both the mRNA expression of three pro-inflammatory cytokines of TNF-α, IL-1β and IL-6 in cells and the same cytokines in cell culture medium decrease, and their expressions are in an order of free Cur > C11G3-TBP > C11G3-TBP@Cur. Meanwhile, we also examined the anti-inflammatory cytokines (M2-type macrophage marker) of activated MH-S cells treated with different materials (Figure 3E). As opposed to the positive control of LPS-treated cells with low mRNA expressions of Arg-1, IL-10 and CD206, the mRNA expressions of these three anti-inflammatory cytokines increase and follow an order of C11G3-TBP@Cur > C11G3-TBP ≈ free Cur. Further, flow cytometry reveals the lower CD86 (typical M1-type marker) expression level and higher Arg-1 (typical M2-type marker) expression level of alveolar macrophages after 24 h incubation with C11G3-TBP@Cur than those treated with the C11G3-TBP or free Cur (Figure S17). The M2 polarization ratio in the C11G3-TBP@Cur group can be calculated to be 59.2%, which is the highest among all groups. These persuasive data demonstrate that C11G3-TBP@Cur could more significantly promote the polarization of alveolar macrophages toward M2 type than drug-free C11G3-TBP and free Cur. Our data strongly suggest that the drug-free C11G3-TBP micelles own an inherent anti-inflammatory activity, which is greater than the drug Cur. Apparently, the C11G3-TBP@Cur micelles combined with both the micelles themselves and Cur have the highest anti-inflammatory activity among the groups.

Furthermore, we also checked the expression of inflammatory mediator NO in cell culture medium and the mRNA level of inducible nitric oxide synthase (iNOS) in the cells, both of which play important roles in accelerating the inflammatory process. Both the NO level in cell culture medium (Figure 3F) and the iNOS mRNA expression (Figure S18) decrease and have an order of free Cur > C11G3-TBP > C11G3-TBP@Cur, similar to the inhibition of pro-inflammatory cytokines (Figure 3D). Hence, the C11G3-TBP@Cur micelles display greater anti-inflammatory activity than the drug-free micelles and free Cur through inhibition of pro-inflammatory cytokines and inflammatory mediator and simultaneous promotion of macrophage repolarization to M2 type.

**Figure 4:**
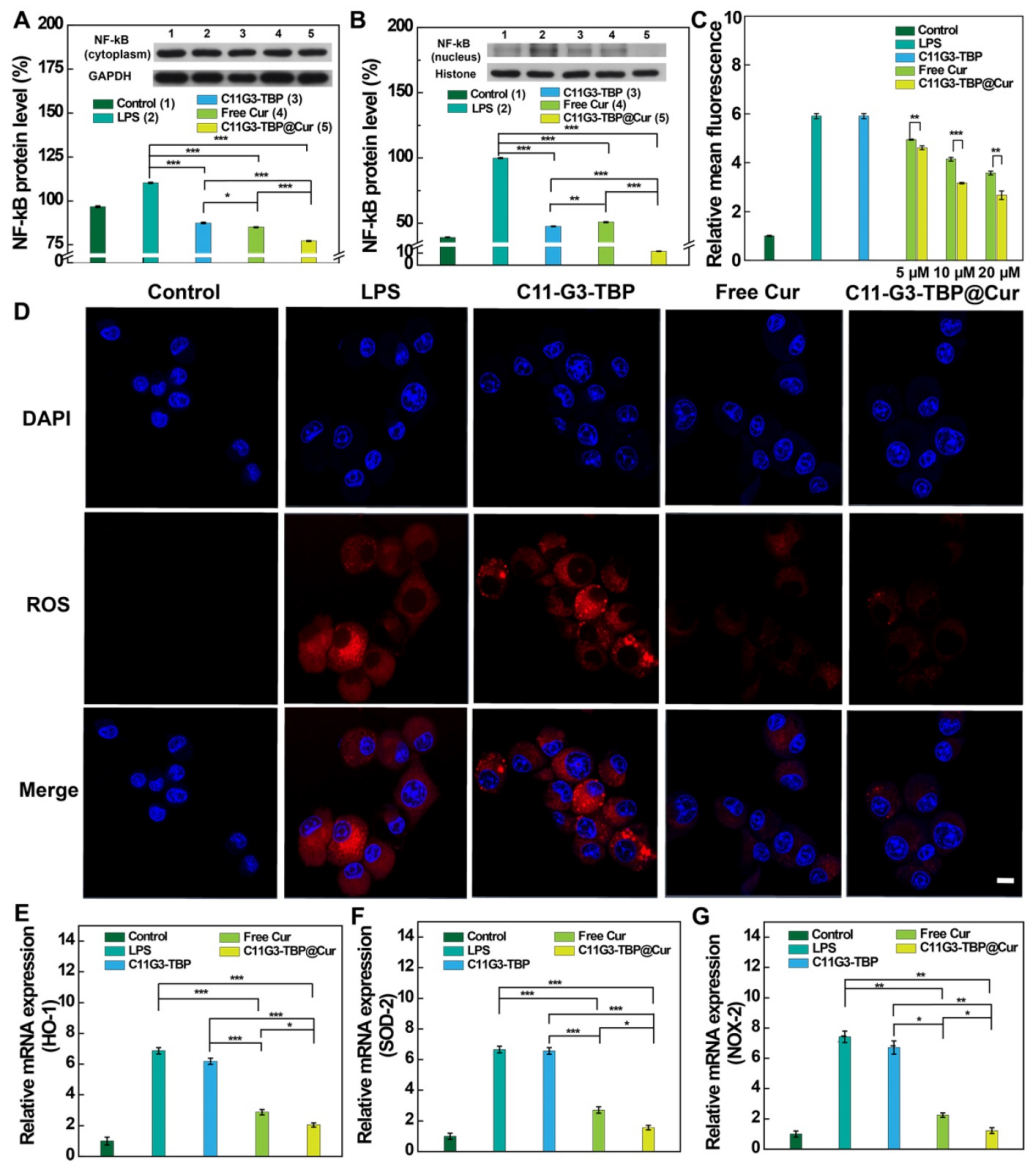
Protein expression levels of NF-κB in cytoplasm (A) and cell nuclei (B) of MH-S cells determined by Western blotting and the quantified expression level (%) of NF-κB relative to β-actin or histone (n = 3). ROS scavenging in LPS-activated MH-S cells after different treatments as tested by (C) flow cytometry and (D) confocal microscopy ([Cur = 10 μM]). In (C), the fluorescence intensity of control group was set to be 1. The scale bar in (D) represents 10 μm. RT-PCR assay of the mRNA expression of antioxidative factors of (E) HO-1, (F) SOD-2 and (G) NOX-2, respectively. Normal MH-S cells were used as negative control, and LPS-activated MH-S cells were used as positive control (the concentration of LPS is 2 μg/mL).

To further validate the anti-inflammatory mechanism of drug-free C11G3-TBP micelles, we next examined the regulatory role of NF-κB in pro-inflammatory cytokine expression (Figure 4A-B). As revealed by Western blotting, cells treated with free Cur, C11G3-TBP, or C11G3-TBP@Cur display reduced NF-κB expression in both the cytoplasm and cell nuclei when compared to the positive LPS control (p < 0.001). Apparently, the drug-free C11G3-TBP micelles have the same NF-κB inhibition effect as free Cur, and the C11G3-TBP@Cur micelles exhibit a combined effect of these two components, thereby efficiently inhibiting the NF-κB expression, especially in the cell nuclei.

### Antioxidative therapy of alveolar macrophages *in vitro*

Cur is reported to exert antioxidative therapy of dysfunctional alveolar macrophages through highly efficient ROS elimination 38. The ROS level in activated MH-S cells after being treated with C11G3-TBP@Cur, C11G3-TBP or free Cur was next checked *via* flow cytometry assay and confocal microscopy imaging. Cells were stained with a ROS probe, ROS Brite™ 670 emitting red fluorescence signals before assays. Flow cytometry assay reveals that the ROS level in MH-S cells in different treatment groups is in an order of LPS control ≈ free C11G3-TBP > free Cur > C11G3-TBP@Cur (Figure 4C). Likely due to the enhanced water solubility and bioavailability of Cur, the C11G3-TBP@Cur micelles exert a much more significant antioxidative efficacy than free Cur (p < 0.01) at the same Cur concentrations. Meanwhile, confocal microscopy imaging reveals that as the positive control, LPS-activated cells display prominent ROS production, which is in contrast to the regular macrophage cells that do not have ROS generation (Figure 4D). Combined with quantitative flow cytometry assay result (Figure 4C) and confocal microscopy imaging fluorescence intensity analysis (Figure S19), the treatment of C11G3-TBP@Cur leads to more significant ROS elimination than free Cur likely because the encapsulation of Cur by C11G3-TBP micelles renders it with improved water solubility and bioavailability. Similar to the flow cytometry assay, the drug-free C11G3-TBP micelles are unable to eliminate ROS, and the C11G3-TBP micelle-treated cells just exhibit the same ROS-associated red fluorescence intensity as the LPS control.

Moreover, the mRNA expressions of the oxidative factors such as heme oxygenase 1 (HO-1), superoxide dismutase 2 (SOD-2), and NADPH oxidase 2 (NOX-2) were also assessed by RT-PCR using standard literature protocols 39. It is evident from Figure 4E-G that the mRNA expression levels of HO-1, SOD-2, and NOX-2 are much higher in the LPS-activated MH-S cells than in the normal MH-S cells. After being treated with C11G3-TBP, C11G3-TBP@Cur, or free Cur, the mRNA expressions of these factors decrease and follow an order of LPS control ≈ C11G3-TBP > free Cur > C11G3-TBP@Cur. These data well corroborate the above ROS generation results, showing that the C11G3-TBP@Cur micelles display greater antioxidative activity than free Cur.

### *In vivo* combination of anti-inflammatory and antioxidative therapy of ALI

**Figure 5:**
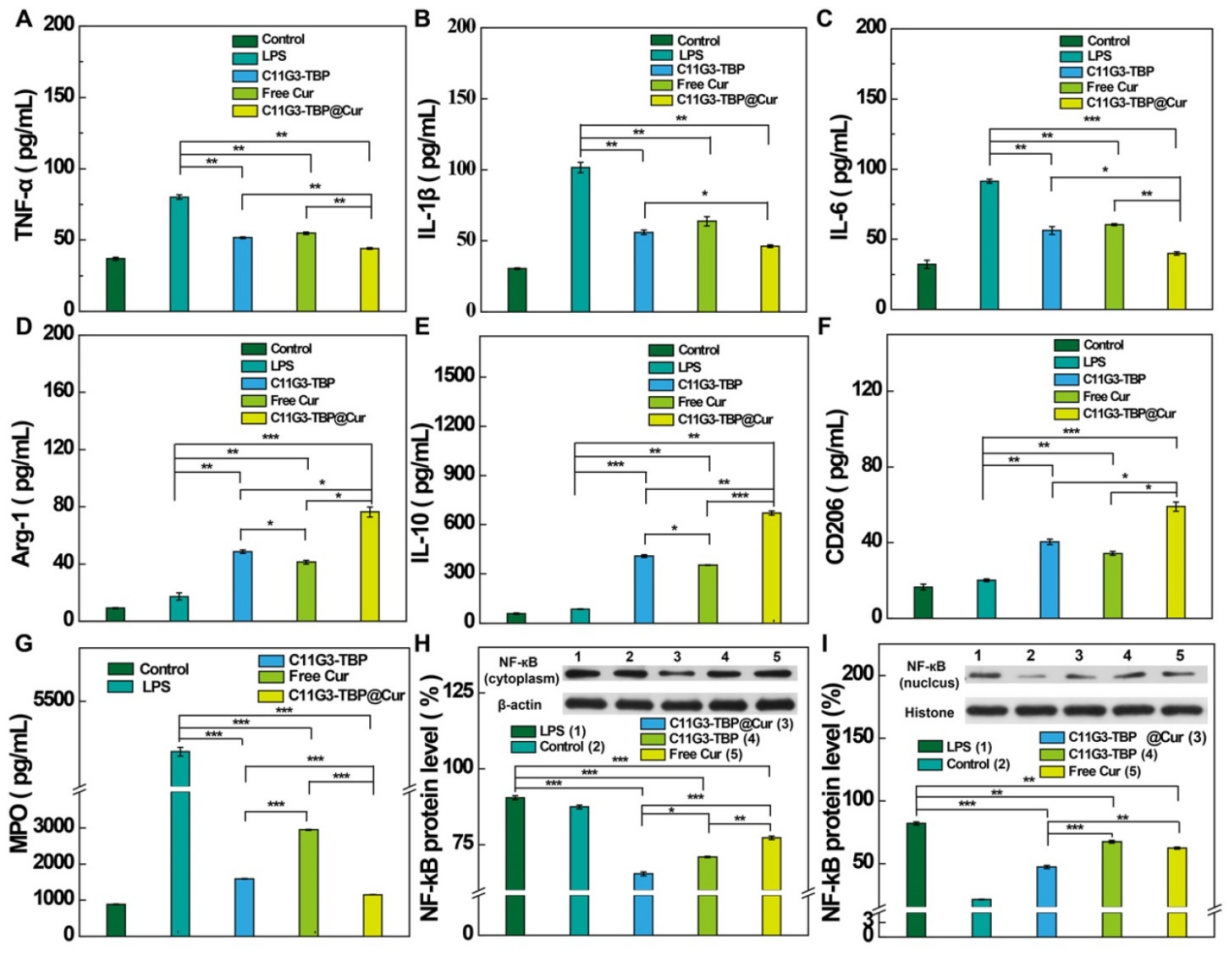
ELISA of the inflammatory cytokine (A) TNF-α, (B) IL-1β, (C) IL-6, (D) Arg-1, (E) IL-10 (F) CD206 and (G) MPO, secretion in BALF in different groups (n = 3). NF-κB protein expressions in cytoplasm (H) and nuclei (I) of injured lung tissue determined by Western blotting and the quantified level (%) of NF-κB relative to β-actin or histone after different treatments (n = 3).

To investigate the *in vivo* therapy effect of C11G3-TBP@Cur nanomicelles for combination ALI treatment, we established an LPS-induced ALI model according to the literature 40. Because systemically administrated NPs exhibit inevitable side effects in major organs and low accumulation in lung lesion, here we used an *in-situ* bronchial atomization administration to treat ALI mice. Since the wet/dry weight ratio of lung is one important indicator of the severity of ALI 41, 42, we first checked this after different treatments. Among all treatment groups (Figure S20), the wet/dry weight ratio of lung tissues is the highest in the LPS-treated positive control group, and decreases after treatments with C11G3-TBP, free Cur or C11G3-TBP@Cur. The treatment of C11G3-TBP@Cur nanomicelles leads to more significantly decreased wet/dry weight ratio than that of C11G3-TBP or free Cur. Next, we checked the inflammatory cytokine secretion levels in the bronchoalveolar lavage fluid (BALF) of each group through ELISA (Figure 5A-F). At 24 h post treatment, the secretion levels of all pro-inflammatory cytokines (TNF-α, IL-1β and IL-6) in the C11G3-TBP@Cur group decline most significantly among all the treatment groups, and follows an order of C11G3-TBP@Cur < C11G3-TBP ≈ free Cur < LPS-treated positive control. Moreover, the secretion levels of all anti-inflammatory cytokines (Arg-1, IL-10 and CD206) in the C11G3-TBP@Cur group are the highest among all the treatment groups, and follows an order of C11G3-TBP@Cur > C11G3-TBP > free Cur > LPS-treated positive control.

As is well known, myeloperoxidase (MPO), an inflammation-related enzyme, is abundant in M1-type macrophages, and high level of MPO leads to effective neutrophils recruitment in the injured lung lesion. Therefore, inhibition of MPO is relevant to the amelioration of the inflammatory storm of lung tissue 41. Hence, we also analyzed the MPO level in the BALF through ELISA (Figure 5G). Notably, following intratracheal atomization administration therapy for 24 h, the treatment of C11G3-TBP@Cur provokes the highest MPO inhibition among all treatment groups, which is close to the negative control. Furthermore, we also measured the mRNA levels of pro-inflammatory and anti-inflammatory cytokines in the lung tissues by RT-PCR (Figure S21). Among all the treatment groups, the treatment of C11G3-TBP@Cur nanomicelles leads to the most significant mRNA downregulation of all pro-inflammatory cytokines and upregulation of all anti-inflammatory cytokines.

In order to confirm the anti-inflammatory therapy mechanism, NF-κB expression in the lung tissues was examined by Western blotting (Figure 5H-I). In consistence with the results of NF-κB expression in alveolar macrophages *in vitro* (Figure 4A-B), the treatment of drug-free C11G3-TBP micelles also leads to significant downregulation of both cytoplasmic and nuclear NF-κB. The NF-κB inhibition follows an order of C11G3-TBP@Cur > C11G3-TBP > free Cur > LPS-treated positive control. Due to the ingenious combination of C11G3-TBP and Cur, the treatment of C11G3-TBP@Cur micelles most significantly inhibits the expression of pro-inflammatory cytokines in the injured lung lesions.

**Figure 6:**
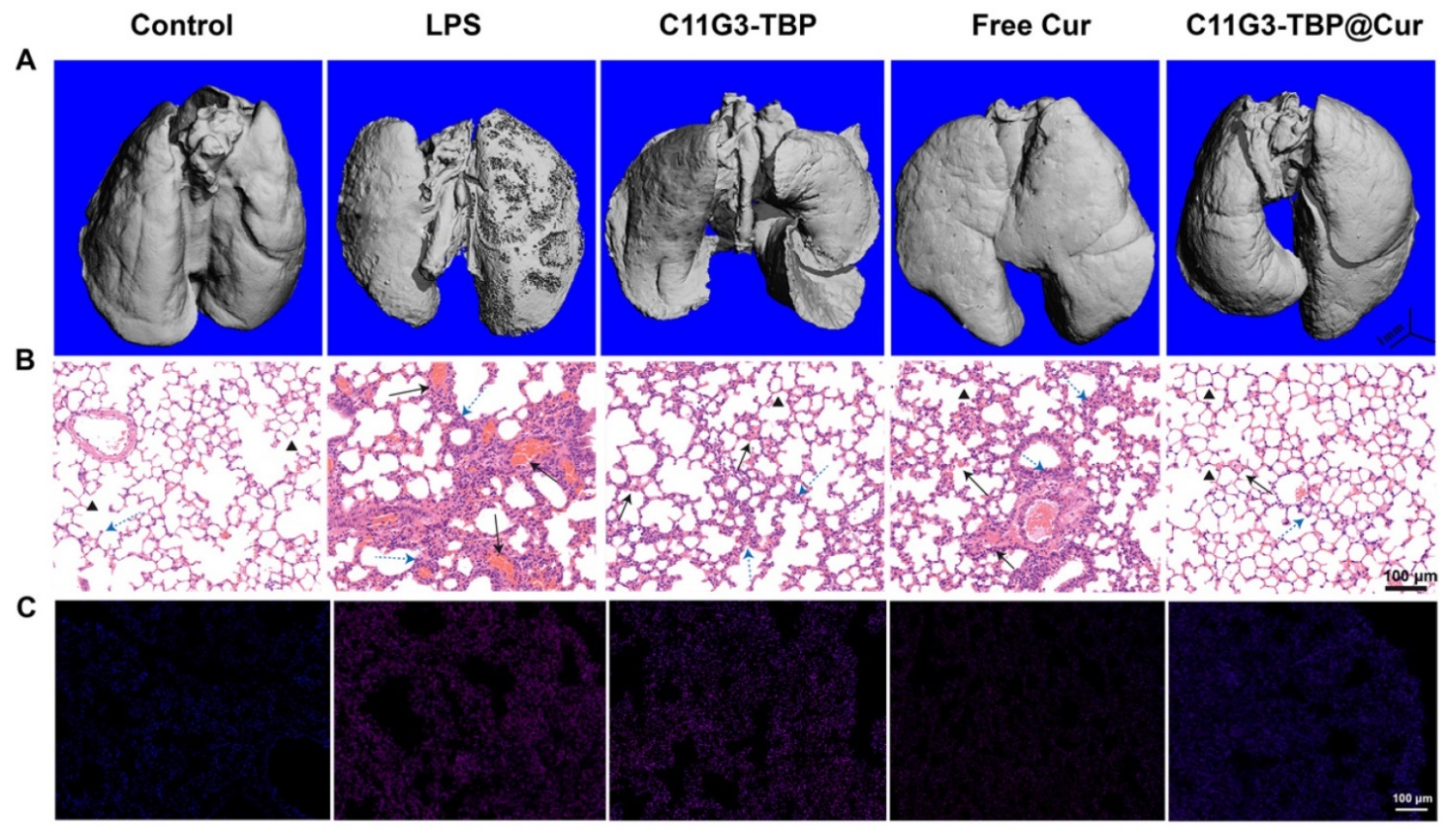
Micro-CT imaging (A), H&E staining (B), and DHE fluorescence probe staining (C) images of the lung tissues extracted from mice of different treatment groups after 24 h treatment. Normal mice administrated with PBS were used as negative control and LPS-induced ALI mice administrated with PBS were used as positive control. In (B), black and blue arrows indicate the alveolar wall congestion and inflammation infiltration, respectively, and black triangles indicate pulmonary alveoli. In (C), red and blue signals represent ROS and cell nuclei, respectively.

Micro-computed tomography (micro-CT) imaging was next used to confirm the excellent combination therapy efficacy of the ALI mice using the C11G3-TBP@Cur nanomicelles (Figure 6A), and the lung tissue erosion in the ALI mice of different treatment groups can be clearly observed. Lung tissue erosion extent for all treatment groups can be quantitatively evaluated by lung tissue volume 43. As expected, the positive control of LPS treatment leads to the smallest lung volume due to the significant lung tissue erosion. Notably, the administration of C11G3-TBP@Cur micelles more significantly recovers back the lung volume in the ALI mice than those of single C11G3-TBP or free Cur (Figure S22), which is quite similar to the normal mice without ALI (negative control).

Furthermore, histological examinations of lung tissues (Figure 6B) and lung injury extent scores (Figure S23) were performed to evaluate the combined anti-inflammatory and antioxidative therapy effect of the C11G3-TBP@Cur nanomicelles in the treatment of ALI mice. Hematoxylin and eosin (H&E) staining of lung tissue slices reveal the serious destruction of alveolar wall and inflammatory infiltration even at the whole lung for the LPS-induced positive control. In contrast, the most efficient lung tissue recovery can be achieved after the C11G3-TBP@Cur treatment, which is featured by reduced hemorrhage of alveolar wall and inflammatory cell infiltration. Likewise, a certain degree of treatment effect can also be observed in the C11G3-TBP and free Cur groups. Correspondingly, in comparison with the negative control group (normal mice), the lung injury scores of all treatment groups are in the order of LPS-treated positive control > free Cur > C11G3-TBP > C11G3-TBP@Cur.

As the ROS level can be used as an indicator to delineate the severity of inflammation, we employed dihydroethidium (DHE) as a fluorescence probe for lung tissue staining (Figure 6C), which can also be quantified through fluorescence intensity measurements (Figure S24). Compared to the negative control, the ROS elimination capacity of lung tissue follows the order of C11G3-TBP@Cur > free Cur > C11G3-TBP ≈ LPS control, verifying the effectiveness of the antioxidative therapy of ALI mainly through the role played by Cur. Similar to the *in vitro* assay (Figure 4C), the drug-free C11G3-TBP micelles do not have any appreciable antioxidant effect. Taken together, the combined anti-inflammatory (dual roles played by both micelles and Cur) and antioxidative (Cur) therapy using the developed amphiphilic phosphorus dendron-based platform integrated with Cur most significantly inhibits the pro-inflammatory cytokine and ROS regulation for effective therapy of lung injury.

Finally, to check the biosafety of the C11G3-TBP@Cur nanomicelles, all the major organs such as heart, liver, spleen and kidney were sectioned and H&E-stained (Figure S25). Obviously, these organs exhibit regular histological morphologies after treatments with free Cur, C11G3-TBP, or C11G3-TBP@Cur in comparison to the control groups, confirming the biocompatibility of the C11G3-TBP@Cur nanomicelles. We further investigated the biodistribution of Cur to assess the metabolization of the C11G3-TBP@Cur nanomicelles at different time points post-administration through fluorescence spectroscopy measurements (Figure S26). Compared to the high Cur level in lung (554.1 μg g^-1^) and very low level of Cur in other organs at 12 h post-administration, the Cur in kidney (119.4 μg g^-1^) and liver (145.5 μg g^-1^) significantly increases at 24 h post-administration, while that in lung (224.1 μg g^-1^) decreases. The descending trend is enhanced with the time post-administtration. These data suggest that the C11G3-TBP@Cur nanomicelles can be cleared *via* liver and kidney, and the retention time of the nanomicelles in the lung tissue is sufficiently long to exert their therapeutic efficacy. Furthermore, blood of the mice after intratracheal atomization administration by C11G3-TBP, free Cur or C11G3-TBP@Cur for 24 h was collected to determine the blood routines including white blood cells (WBC), percentage of monocytes (Mon%) and percentage of neutrophil granulocyte (Gran%). As opposed to the LPS-treated positive control, all treatment groups show the normal range of the respective indicators (Figure S27), further elaborating the good biosafety profiles of the C11G3-TBP@Cur nanomicelles.

## Conclusions

In summary, we developed unique bisphosphonate-modified amphiphilic phosphorus dendron micelles to encapsulate Cur for combination anti-inflammatory and antioxidative therapy of ALI. The C11G3-TBP dendrons bearing a long linear alkyl chain (C_11_H_23_) as cores, rigid phosphorous branches and eighty bisphosphonate terminal groups can be self-assembled to form nanomicelles with a size of 91.1 nm for highly efficient loading of hydrophobic drug Cur with a loading percentage of 21.24% and encapsulation efficiency of 96.86%. The thus created C11G3-TBP@Cur nanomicelles possess good colloidal stability and cytocompatibility, can effectively scavenge ROS and inhibit the pro-inflammatory cytokine (TNF-α, IL-1β and IL-6) regulation through blocking of NF-κB signal pathway in M1-type alveolar macrophages, and enable effective repolarization of alveolar macrophages to anti-inflammatory M2 type to boost combination anti-inflammatory and antioxidative therapy of LPS-activated alveolar macrophages *in vitro* and a mouse ALI model *in vivo*. Most strikingly, we show that the drug-free C11G3-TBP nanomicelles display inherent anti-inflammatory activity to inhibit the expression of NF-κB, and the combination with Cur in a formulation of C11G3-TBP@Cur nanomicelles can fully take advantages of each party to boost combination therapy of ALI through downregulation of NF-κB and pro-inflammatory cytokines and elimination of ROS in the ALI lesion, thereby causing significant inhibition of inflammation infiltration and alveolar wall damage of injured lung tissues. The created original phosphorus dendron-based nanomicelles may represent one of the most updated nanomedicines for effective ALI therapy or other inflammatory diseases with a promising potential for clinical translation.

## Experimental Section

**Materials.** All chemicals and reagents were available from commercial sources, and all solvents were routinely dried and distilled before use. LPS was acquired from Sigma-Aldrich (St. Louis, MO). MH-S cells (a mouse alveolar macrophage cell line), Roswell Park Memorial Institute 1640 (RPMI 1640) medium, fetal bovine serum (FBS) and β-mercaptoethanol were supplied from Zhong Qiao Xin Zhou Biotechnology Co., Ltd. (Shanghai, China).

**Preparation of anionic phosphorus dendron-based nanomedicine C11G3-TBP@Cur.** Cur (ranging from 1.54 to 3.85 mg) dissolved in 300 μL methanol was mixed with C11G3-TBP (10 mg) in 3.0 mL water at different molar ratios (C11G3-TBP: Cur = 1: 10, 1: 15, 1: 20 or 1:25). Each mixture was magnetically stirred at room temperature overnight to leave the volatilization of methanol, and then centrifuged. The supernatant was lyophilized to obtain the C11G3-TBP@Cur nanomicelles.

**Pro-inflammatory cytokine expression *in vitro*.** MH-S cells were seeded, activated with LPS for 24 h, and treated with drug-free C11G3-TBP, C11G3-TBP@Cur or free Cur under different concentrations. After 24 h cultivation, culture medium of each well was collected to analyze the pro-inflammatory factors (TNF-α, IL-1β and IL-6) using commercial ELISA kits, and inflammatory mediator NO using commercial Griess Reagent kits. The cells were collected to determine the inflammatory factor-related mRNA using RT-PCR according to the manufacturer's protocols by referring to the *β-actin* gene.

**NF-κB transcription factor assay* in vitro*.** MH-S cells were seeded, activated with LPS for 24 h, and treated with drug-free C11G3-TBP, C11G3-TBP@Cur or free Cur at a Cur or equivilent Cur concentrtaion of 10 μM and cultivated for 24 h. Extraction of cytoplasmic or nuclear NF-κB proteins from MH-S cells was carried out using commercial Nuclear and Cytoplasmic Protein Extraction Kit. The obtained nuclear proteins and cytoplasmic proteins were used to detect NF-κB through Western blotting according to the literature protocols 44.

**Oxidative stress assay.** MH-S cells were seeded, LPS-activated, treated with C11G3-TBP@Cur or free Cur ([Cur] = 5-20 μM) for 6 h, and incubated with ROS detection agent (ROS Brite™ 670) and DAPI, respectively. After the staining process, the cells were quantitatively analyzed by flow cytometry and observed by laser scanning confocal microscopy, respectively. To further reveal the antioxidative property of C11G3-TBP@Cur, the antioxidative-related mRNAs (HO-1, SOD-2 and NOX-2) were examined using RT-PCR under the protocols mentioned above.

***In vivo* ALI therapy.** All animal experiments were approved by and carried out according to the guidelines of the Committee on Experimental Animal Care and Use of Donghua University and also following the regulations of the National Ministry of Health. Male Balb/c mice were intraperitoneally injected with LPS solution to induce ALI. At 24 h post LPS challenge, free Cur (5 mg/kg, 100 μL PBS (1% DMSO)), C11G3-TBP (6.48 mg/kg, 100 μL PBS), and C11G3-TBP@Cur (21.83 mg/kg, 100 μL PBS) were respectively aerosolized and inhaled by each mouse. After 24 h, animals were sacrificed to obtain lung tissue and BALF. Lung tissues were collected, and the wet/dry weight ratio was calculated to evaluate the anti-inflammatory effect of lung tissue. BALF was centrifuged to collect the supernatant for quantification of pro-inflammatory factors (TNF-α, IL-1β and IL-6), anti-inflammatory factors (Arg-1, IL-10 and CD206) and neutrophils infiltration marker MPO using commercial ELISA kits. In addition, the parallel lung tissues were homogenized to collect total RNA *via* a Trizol reagent for RT-PCR determination of the mRNA expression levels of pro-inflammatory factors (TNF-α, IL-1β and IL-6) and anti-inflammatory factors (Arg-1, IL-10 and CD206). The assay was carried out according to the aforementioned protocols and *GAPDH* was employed as a reference gene. Further, the homogenized lung tissues were treated with Nuclear and Cytoplasmic Protein Extraction Kit to determine the nuclear and cytoplasmic NF-κB protein content through Western blotting 44.

**Statistical analysis.** A one-way analysis of variance statistical method was adopted to evaluate the significance of the data for comparison of in-between groups using IBM SPSS Statistic 26 software (IBM, Armonk, NY). A value of 0.05 was considered as the level of significance, and the associated data were indicated as (*) for p < 0.05, (**) for p < 0.01, (***) for p < 0.001, respectively. See full experimental details in the Supporting Information.

## Supplementary Material

Supplementary materials and methods, figures.
