# Supplementary material for: Phosphorus dendron nanomicelles as a platform for combination anti-inflammatory and antioxidative therapy of acute lung injury
Source: Theranostics. 2022 Apr 11;12(7):3407–19. doi: 10.7150/thno.70701 (PMC9065184; doi:10.7150/thno.70701)
Supplement: Supplementary file 1 — Supplementary materials and methods, figures. [file thnov12p3407s1.pdf]

Supporting information

Phosphorus dendron nanomicelles as a platform for combination anti-inflammatory and antioxidative therapy of acute lung injury

Jin Li[1], Liang Chen[1], Changsheng Li[1], Yu Fan[1], Mengsi Zhan[1], Huxiao Sun[1], Serge Mignani[4,5], Jean-Pierre Majoral[2,3], Mingwu Shen[1], Xiangyang Shi[1,5]**

[1] State Key Laboratory for Modification of Chemical Fibers and Polymer Materials, Shanghai Engineering Research Center of Nano-Biomaterials and Regenerative Medicine, College of Chemistry, Chemical Engineering and Biotechnology, Donghua University, Shanghai 201620, People's Republic of China

[2] Laboratoire de Chimie de Coordination du CNRS, 205 Route de Narbonne, BP 44099, 31077 Toulouse CEDEX 4, France

[3] Université de Toulouse, UPS, INPT, 31077 Toulouse CEDEX 4, France

[4] Université Paris Descartes, PRES Sorbonne Paris Cité, CNRS UMR 860, Laboratoire de Chimie et de Biochimie Pharmacologiques et Toxicologique, 45, rue des Saints Pères, 75006 Paris, France

[5] CQM-Centro de Quimica da Madeira, Universidade da Madeira, 9020-105 Funchal, Portugal

* Corresponding author. E-mail: mwshen@dhu.edu.cn (M. W. Shen) and xshi@dhu.edu.cn (X. Y. Shi)

Experimental Section

Materials. All chemicals and reagents were available from commercial sources, and all solvents were routinely dried and distilled before use. Curcumin (Cur) was from J&K Scientific Ltd. (Beijing, China). 1-(3-Dimethylaminopropyl)-3-ethylcarbodiimide hydrochloride (EDC·HCl) was from Sinopharm Chemical Reagent Co., Ltd. (Shanghai, China). Tween-80 was from Titan Scientific Co., Ltd. (Shanghai, China). MH-S cells (a mouse alveolar macrophage cell line), Roswell Park Memorial Institute 1640 (RPMI 1640) medium, fetal bovine serum (FBS) and β-mercaptoethanol were supplied from Zhong Qiao Xin Zhou Biotechnology Co., Ltd. (Shanghai, China). Penicillin and streptomycin were from Gino Biomedical Technology Co., Ltd. (Hangzhou, China). Cell Counting Kit-8 (CCK-8), BeyoRTM first strand cDNA synthesis kit, total RNA extractor (Trizol), Beyo FastTM SYBR Green qPCR Mix (2×, Low ROX), Bradford protein assay kit, Griess Reagent kits, Nuclear and Cytoplasmic Protein Extraction kit, Western blot kit with HRP-labeled goat anti-mouse IgG (H+L), and 4',6-diamidino-2-phenylindole (DAPI) were from Beyotime Biotechnology Co., Ltd. (Shanghai, China). Enzyme Linked Immunosorbent Assay kits (*TNF-α, IL-1β, IL-6, and MPO*) were from (Multisciences Biotechnology, Hangzhou, China). Enzyme Linked Immunosorbent Assay kits (*Arg-1, CD206 and IL-10)*, rabbit primary polyclonal antibodies of anti-NF-κB, β-actin and histone were from Servicebio (Wuhan, China). APC-CD86 and PE-Arg-1 monoclonal antibodies were from Thermo Fisher Scientific (Waltham, MA). The primers (Table S1) of inflammatory (*TNF-α, IL-1β, IL-6, IL-10, CD206 and Arg-1*), antioxidation factors (*HO-1, SOD-2, and NOX-2*), reference genes (*β-actin* and *GADPH*) and inducible nitric oxide synthase (iNOS) were from Generay (Shanghai, China). Cell Meter™ Fluorimetric Intracellular Total ROS Activity Assay Kit was acquired from Biolite Biotech Co., Ltd. (Xi'an, China). Dihydroethidium (DHE) superoxide anion fluorescent probe and lipopolysaccharide (LPS) were acquired from Sigma-Aldrich (St. Louis, MO). Water used in all experiments was purified using a PURIST UV Ultrapure Water System (RephiLe Bioscience, Ltd., Shanghai, China) with a resistivity higher than 18.2 MΩ·cm.

NMR characterization. ^{1}H and ^{31}P NMR spectra were recorded with Bruker AV400 NMR

spectrometer (Fällanden, Switzerland). All ^{31}P NMR spectra were generally recorded by avoiding the disturbance of {^{1}H}. The structures of all synthesized compounds are shown in Figure S1.

Compound 1: This compound was prepared and characterized according to the literature [1]. The solution of hexachlorocyclotriphosphazene (17.25 mmol, 50 mL tetrahydrofuran (THF)) was added into the mixture of 4-hydroxybenzaldehyde (86.25 mmol) and anhydrous potassium carbonate (172.5 mmol) in 50 mL THF under an ice bath for 20 min. The reaction mixture was stirred for 12 h at room temperature. Salts were then removed by centrifugation and the clear solution was concentrated under reduced pressure. The residue was then purified by silica column chromatography (hexane/ethyl acetate, 8/2 to 6/4, v/v) to afford compound 1 as a colorless oil in 76% yield. ^{1}H NMR (400 MHz, MeOD): δ= 7.24 (m, 10 H, C_0^2-H), 7.82 (m, 10 H, C_0^3-H), 9.98 (t, $^4J_{(H\text{-}C\text{-}C13\text{-}H)}$ =4Hz, 5H, CHO) ppm. ^{31}P{^{1}H} NMR (121 MHz, CDCl3): δ= 5.19 (d, $^2J_{(p\text{-}p)}$ =84 Hz, P_{01}, P_{02}), 20.73 (dd, $^2J_{P\text{-}P}$ = 88 Hz, $^2J_{P\text{-}P}$ = 84 Hz, P_{03}) ppm.

Compound 2: The solution of the tyramine (1.82 mmol, 5 mL methanol (MeOH)) was added into the activated lauric acid (1.82 mmol) by EDC·HCl (1.82 mmol) in 10 mL dichloromethane (DCM). This reaction mixture was refluxed for 12 h and then concentrated under reduced pressure. The gross product was then purified by silica column chromatography (DCM/MeOH = 19/1, v/v) to afford compound 2 as a pale yellow powder in 80.2% yield. ^{1}H NMR (400 MHz, MeOD): δ= 0.91 (t, $^3J_{(H\text{-}H)}$ =8 Hz, 3H, C_{0}^{i}-H), 1.31 (m, 16 H, C_{0}^{h}-H, C_{0}^{g}-H and C_{0}^{f}-H), 1.60 (m, 2 H, C_{0}^{e}-H), 2.15 (t, $^3J_{(H\text{-}H)}$ =8 Hz, 2 H, C_{0}^{d}-H), 2.70 (t, $^3J_{(H\text{-}H)}$ =8 Hz, 2 H, C_{0}^{a}-H), 3.36 (m, 2 H, C_{0}^{b}-H), 4.88 (HDO), 6.72 (d, $^3J_{(H\text{-}H)}$ =8 Hz, 2 H, C_{0}^{2}-H), 7.04 (d, $^3J_{(H\text{-}H)}$ =8 Hz, 2 H, C_{0}^{3}-H) ppm.

Compound 3: According to the literature [2], the solution of thiophosphoryl chloride (30.7 mmol, 100 mL chloroform ($CHCl_3$)) was dropwise added into the solution of methylhydrazine (61.4 mmol, 10 mL $CHCl_3$) at -61 °C under an acetone/liquid nitrogen slurry bath. The reaction mixture was stirred overnight at room temperature and then reactants were filtered to obtain the methylhydrazine-modified thiophosphoryl (compound 3) in 91% yield.

Compound 4: The solution of compound 2 (0.495 mmol, 10 mL THF) was added into the mixture of cesium carbonate (0.99 mmol) and compound 1 (0.33 mmol) in THF (10 mL) at 0 °C. The reaction

mixture was stirred for 12 h at room temperature and then centrifuged (8000 rpm, 10min). The supernatant was then concentrated under reduced pressure. Crude product was purified by silica column chromatography (hexane/ethyl acetate, 6/4, v/v) to afford compound 4 as a white powder in 85.1% yield. ^{1}H NMR (400 MHz, $CDCl_3$): δ= 0.87 (t, $^{3}J_{(H\text{-}H)}$=8 Hz , 3 H, C^{i}_{0}-H), 1.30 (m, 16 H, C^{h}_{0}-H, C^{g}_{0}-H and C^{f}_{0}-H), 1.59 (m, 2 H, C^{e}_{0}-H), 2.13 (t, $^{3}J_{(H\text{-}H)}$=8 Hz, 2 H, C^{d}_{0}-H), 2.79 (t, $^{3}J_{(H\text{-}H)}$=8 Hz, 2 H, C^{a}_{0}-H), 3.47 (m, 2 H, C^{b}_{0}-H), 5.67 (t, $^{3}J_{(H\text{-}H)}$=6 Hz, 1 H, NH), 6.93 (d, $^{3}J_{(H\text{-}H)}$= 8 Hz, 2 H, C^{2}_{0}-H), 7.04 (d, $^{3}J_{(H\text{-}H)}$=8 Hz, 2 H, C^{3}_{0}-H), 7.13 (m, 10 H, C^{2}_{0}-H), 7.74 (t, $^{3}J_{(H\text{-}H)}$=8 Hz, 10 H, C^{3}_{0}-H), 9.94 (m,5 H, CHO) ppm. ^{31}P NMR (162 MHz, $CDCl_3$): δ= 7.40 (m, P_3N_3) ppm.

Compound 5: The freshly prepared solution of compound 3 (2.10 mmol, 10 mL $CHCl_3$) was added into the mixture of compound 4 (0.35 mmol) and anhydrous sodium sulfate (4.20 mmol) in DCM (50 mL) at 0 °C. The reaction mixture was stirred for 6 h at room temperature. Salts were removed by filtration and then the clear solution was concentrated under reduced pressure. The residue was added into 100 mL pentane and stirred for 30 min. The resulting precipitate was filtered off and dried under reduced pressure to afford compound 5 as a colorless powder in 90.1% yield. ^{1}H NMR (400 MHz, $CDCl_3$): δ=0.89 (t, $^{3}J_{(H\text{-}H)}$=8 Hz, 3 H, C^{i}_{0}-H), 1.27 (m, 16 H, C^{g}_{0}-H, C^{h}_{0}-Hand C^{f}_{0}-H), 1.59 (m, 2 H, C^{e}_{0}-H), 2.12 (t, $^{3}J_{(H\text{-}H)}$=8 Hz, 2 H, C^{d}_{0}-H), 2.77 (t, $^{3}J_{(H\text{-}H)}$=8 Hz, 2 H, C^{a}_{0}-H), 3.44 (m, 2 H, C^{b}_{0}-H), 3.50 (m, 15 H, CH_3-N-P_1), 5.47 (t, $^{3}J_{(H\text{-}H)}$=8 Hz, 1 H, NH), 6.92 (d, $^{3}J_{(H\text{-}H)}$= 12 Hz, 2 H, C^{2}_{0}-H), 7.03 (m, 10 H, C^{2}_{0}-H), 7.61 (m, 15 H, C3 0, CH=N), 7.70 (br s, 2 H, C^{3}_{0}-H) ppm. ^{31}P NMR(162 MHz, $CDCl_3$): δ= 8.28 (m, P_3N_3), 62.40, 62.44 (s, P_1) ppm.

Compound 6: This compound was prepared and characterized according to the literature [2]. The solution of 4-hydroxybenzaldehyde (7 mmol, 5 mL THF) was added into the mixture of thiophosphoryl chloride (3.5 mmol) and cesium carbonate (35.1 mmol) in THF (10 mL) at -80 °C under an acetone/liquid nitrogen slurry bath. The reaction mixture was stirred overnight at room temperature and then centrifuged (8000 rpm, 10min). The supernatant was concentrated under reduced pressure. Crude product was purified by silica column chromatography (hexane/ethyl acetate, 9/1, v/v) to afford compound 6 as a white powder in 80.4% yield.

Compound 7: This compound was prepared and characterized according to the literature [2]. The

solution of sodium azide (3.5 mmol, 2 mL THF) was added into the solution of compound 6 (2.94 mmol) in 10 mL THF at 0 °C. The reaction mixture was stirred for 12 h at room temperature and then filtered. The supernatant was concentrated under reduced pressure. The resulting product was purified by silica column chromatography (hexane/ethyl acetate, 7/3, v/v) to afford compound 7 as a white powder in 92.4% yield.

Compound 8: According to the literature,[2] diphenylphosphine (1 mmol) was dissolved in 5 mL oxygen-free DCM at 0 °C and then 4-lodophenol (1 mmol, 2 mL oxygen-free DCM) was dropwise added. The mixture was stirred for 3 h at 130 °C and then vacuum dried to obtain compound 8 as a white powder in 90.2% yield.

Compound 9: According to the literature [2], the solution of compound 8 (2 mmol, 5 mL oxygen-free DCM) was added into compound 7 (2 mmol, 10 mL oxygen-free DCM) at 0 °C, and the reaction mixture was stirred for 12 h at room temperature, purified by silica column chromatography (hexane/ethyl acetate, 1.5/1, v/v), and then vacuum dried to afford compound 9 as a white powder in 92.8% yield.

Compound 10: Compound 5 (0.1 mmol) and cesium carbonate (1 mmol) were dissolved in 10 mL anhydrous DCM at 0 °C, and then compound 10 (0.5 mmol, 5 mL anhydrous DCM) was dropwise added. The reaction mixture was stirred for 12 h at room temperature. The resulting precipitate was filtered off and then crude product was concentrated under reduced pressure. Crude product was dissolved in 10 mL anhydrous DCM, and 100 mL mixture (pentane/diethyl ether, 8/2, v/v) was dropwise added. The mixture was stirred for 0.5 h at room temperature and then vacuum dried to obtain compound 10 as a white powder in 91.1% yield. ^{1}H NMR(400 MHz, $CDCl_3$): δ=0.85 (t, $^{3}J_{(H\text{-}H)}$ =8 Hz , 3 H, $C^{i}_{0'}$-H), 1.22 (m, 18 H, $C^{g}_{0'}$-H, $C^{h}_{0'}$-H and $C^{f}_{0'}$-H), 1.96 (t, $^{3}J_{(H\text{-}H)}$ =8 Hz, 2 H, $C^{d}_{0'}$-H), 2.60 (t, $^{3}J_{(H\text{-}H)}$ =8 Hz, 2 H, $C^{a}_{0'}$-H), 2.80 (m, 2 H, $C^{b}_{0'}$-H), 3.32 (m, 15 H, CH_3-N-P_1), 5.98 (t, $^{3}J_{(H\text{-}H)}$ =6 Hz, 1 H, NH), 6.94 (d, $^{3}J_{(H\text{-}H)}$ = 8 Hz, 2 H, $C^{2}_{0'}$-H), 7.09 (m, 12 H, $C^{3}_{0'}$-H and C^{0}_{2}-H), 7.26 (m, 40 H, C^{2}_{2}-H), 7.42 (m, 50 H, C^{3}_{0}-H, C^{2}_{1}-H and C^{3}_{1}-H), 7.58(m, 105 H, C^{6}_{1}-H, C^{7}_{1}-H, C^{8}_{1}-H and CH=N), 7.75 (m, 40 H, C^{3}_{2}-H), 9.86 (m,20 H, CHO) ppm. ^{31}P NMR (162 MHz, $CDCl_3$): δ=7.84 (m, P_3N_3), 13.75 (d, $^{2}J_{(PN\text{-}P2)}$ =31 Hz, PN), 49.81 (td, $^{2}J_{(P2\text{-}PN)}$ =31 Hz, P_2), 60.49, 60.58 (s, P_1) ppm.

Compound 11: Compound 10 (0.05 mmol) and cesium carbonate (0.5 mmol) were dissolved in 10 mL DCM at 0 °C. Compound 3 (1 mmol, 5 mL DCM) was dropwise added and the reaction mixture was stirred for 6 h at room temperature. The resulting precipitate was filtered off and then crude product was concentrated under reduced pressure. Crude product was dissolved in 10 mL anhydrous THF, and 100 mL pentane was dropwise added. The mixture was stirred for 0.5 h at room temperature and then vacuum dried to obtain compound 11 as a white powder in 92.4% yield. ^{1}H NMR (400 MHz, $CDCl_3$): δ=0.85 (t, $^{3}J_{(H\text{-}H)}$=8 Hz , 3 H, $C^{i}_{'0}$-H), 1.19 (m, 18 H, $C^{e}_{'0}$-H, $C^{f}_{'0}$-H, $C^{h}_{'0}$-H and $C^{g}_{'0}$-H), 1.98 (t, $^{3}J_{(H\text{-}H)}$=8 Hz, 2 H, $C^{d}_{'0}$-H), 2.57 (t, $^{3}J_{(H\text{-}H)}$=8 Hz, 2 H, $C^{a}_{'0}$-H), 3.25 (m, 2 H, $C^{b}_{'0}$-H), 3.32 (m, 15 H, CH_3-N-P_1), 3.45 (m, 60 H, CH_3-N-P_3), 5.91 (t, $^{3}J_{(H\text{-}H)}$=4 Hz, 1 H, NH), 6.90 (d, $^{3}J_{(H\text{-}H)}$= 8 Hz, 2 H, $C^{2}_{'0}$-H), 7.05 (m, 12 H, $C^{3}_{'0}$-H and C^{0}_{2}-H), 7.15 (m, 40 H, C^{2}_{2}-H), 7.29 (m, 10 H, C^{3}_{0}-H), 7.39 (m, 40 H, C^{3}_{2}-H), 7.60 (m, 165 H, C^{2}_{1}-H, C^{3}_{1}-H, C^{6}_{1}-H, C^{7}_{1}-H, C^{8}_{1}-H, CH=N) ppm. ^{31}P NMR (162 MHz, $CDCl_3$): δ=7.76 (m, P_3N_3), 13.16 (d, $^{2}J_{(PN\text{-}P2)}$=30 Hz, PN), 50.95 (td, $^{2}J_{(P2\text{-}PN)}$=30 Hz, P_2), 60.70, 60.79 (s, P_1), 62.96 (s, P_3) ppm.

Compound 12: Tyramine (51.2 mmol) was dissolved in THF (50 mL) at 0 °C. The formaldehyde (37% in water, 8 mL, 104 mmol) was dropwise added. The resulting mixture was stirred for 30 min at room temperature and then dimethylphosphite (10 mL, 110 mmol) was added. The mixture was stirred at room temperature for 24 h, and added with 50 mL of brine to extract the product three times with 200 mL of ethyl acetate. The extracted liquid was added with magnesium sulphate and then concentrated under reduced pressure. The resulting crude oil was purified by silica column chromatography (acetone) to afford compound 12 as a colorless oil in 65% yield. ^{1}H NMR (400 MHz, $CDCl_3$): δ = 2.71 (m, 2H, C_5-H); 3.04 (m, 2H, C_6-H); 3.21 (d, $^{2}J_{HP}$ = 9.0 Hz, 4H, C_7-H), 3.78 (d, $^{3}J_{HP}$ =10.6 Hz, 12H, OMe); 6.79 (m, 2H, C^2-H); 7.01 (m, 2H, C^3-H); 8.46 (brs, 1H, OH). ^{31}P NMR (162 MHz, $CDCl_3$): δ= 27.21 (s, PO_3Me_2)

Compound 13: Compound 11 (9.4 μmol), Compound 12 (0.38 mmol) and cesium carbonate (0.75 mmol) were co-dissolved in anhydrous THF (10 mL) at 0 °C. The mixture was stirred at room temperature for 24 h and then centrifuged (8000 rpm, 10 min). The supernatant was concentrated under reduced pressure. The resulting crude oil was dissolved in anhydrous THF (10 mL) and dropwise

added to 100 mL hexane/ethyl acetate (7/3, v/v). The mixture was finally evaporated under reduced pressure to afford compound 13 as a colorless oil in 85% yield. ^{1}H NMR (400 MHz, $CDCl_3$): δ=0.87 (t, $^{3}J_{(H\text{-}H)}$=8 Hz , 3 H, C_0^{i}-H), 1.28 (m, 18 H, C_0^{e}-H, C_0^{f}-H, C_0^{h}-H and C_0^{g}-H), 1.90 (t, $^{3}J_{(H\text{-}H)}$=8 Hz, 2 H, C_0^{d}-H), 2.70 (t, $^{3}J_{(H\text{-}H)}$=8 Hz, 2 H, C_0^{a}-H), 3.04 (m, 2 H, C_5-H), 3.18 (m, 2 H, C_0^{b}-H), 3.31 (m, 15 H, CH_3-N-P_1), 3.71 (m, 60 H, CH_3-N-P_3), 3.78 (d, $^{3}J_{HP}$ = 10.6 Hz, 12 H, OMe), 5.71 (t, $^{3}J_{(H\text{-}H)}$=4 Hz, 1 H, NH), 6.79 (m, 2 H, C_3^{2}-H), 6.80 (d, $^{3}J_{(H\text{-}H)}$= 8 Hz, 2 H, C_0^{2}-H), 7.03 (m, 2 H, C_3^{3}-H), 7.04 (m, 12 H, C_0^{3}-H and C_2^{0}-H), 7.16 (m, 40 H, C_2^{2}-H), 7.29 (m, 10 H, C_0^{3}-H), 7.38 (m, 40 H, C_2^{3}-H), 7.63 (m, 165 H, C_1^{2}-H, C_1^{3}-H, C_1^{6}-H, C_1^{7}-H, C_1^{8}-H, CH=N) ppm. ^{31}P NMR (162 MHz, $CDCl_3$): δ=7.67 (m, P_3N_3), 13.03 (d, $^{2}J_{(PN\text{-}P2)}$=30 Hz, PN), 50.95 (td, $^{2}J_{(P2\text{-}PN)}$=30 Hz, P_2), 26.82 (s, P_4), 61.06, 62.12 (s, P_1), 63.31(s, P_3) ppm.

Compound 14: Compound 13 (4.1 μmol) was dissolved in acetonitrile (10 mL) at 0 °C and then trimethylsilylbromide (0.33 mmol) was dropwise added. The reaction mixture was stirred at room temperature overnight and then evaporated to dryness under reduced pressure. The crude product was washed twice with MeOH (5 mL) for 30 min at room temperature and concentrated under reduced pressure. The resulting white solid was washed once with diethylether (20 mL) and then suspended in water (1 mL/100 mg). The equivalent mole of sodium hydroxide (0.2 mol/L) per terminal phosphonic acid was dropwise added. The resulting solution was lyophilized to afford a dendron compound 14, named C11G3-TBP as a white powder in 80% yield. ^{1}H NMR (400 MHz, D_2O/CD_3CN 9:1): δ=0.89 (t, $^{3}J_{(H\text{-}H)}$=8 Hz , 3 H, C_0^{i}-H), 1.29 (m, 18 H, C_0^{e}-H, C_0^{f}-H, C_0^{h}-H and C_0^{g}-H), 1.90 (t, $^{3}J_{(H\text{-}H)}$=8 Hz, 2 H, C_0^{d}-H), 2.50 (t, $^{3}J_{(H\text{-}H)}$=8 Hz, 2 H, C_0^{a}-H), 2.97 (m, 2 H, C_5-H), 3.10 (m, 2 H, C_0^{b}-H), 3.25 (m, 15 H, CH_3-N-P_1), 3.60 (m, 60 H, CH_3-N-P_3), 3.77 (d, $^{3}J_{HP}$ = 10.6 Hz, 12 H, OMe), 6.60 (m, 2 H, C_3^{2}-H), 6.63 (d, $^{3}J_{(H\text{-}H)}$= 8 Hz, 2 H, C_0^{2}-H), 7.12 (m, 2 H, C_3^{3}-H), 7.13 (m, 12 H, C_0^{3}-H and C_2^{0}-H), 7.22 (m, 40 H, C_2^{2}-H), 7.30 (m, 10 H, C_0^{3}-H), 7.39 (m, 40 H, C_2^{3}-H), 7.63(m, 165 H, C_1^{2}-H, C_1^{3}-H, C_1^{6}-H, C_1^{7}-H, C_1^{8}-H, CH=N) ppm. ^{31}P NMR (162 MHz, D_2O/CD_3CN 9:1): δ=8.66 (s, P_4), 9.97 (m, P_3N_3), 11.81 (m, PN), 26.82 (s, P_4), 54.40 (brs, P_2), 64.42 (brs, P_1) ppm.

Critical micellar concentration (CMC) determination. The CMC of C11G3-TBP was assessed using pyrene as a fluorescent probe. A series of C11G3-TBP solutions with concentration

ranging from 4×10^{-8} to 6.1×10^{-5} M were prepared in water. These solutions were respectively added into centrifuge tubes containing the fluorescent probe pyrene with a final concentration of 4.0×10^{-4} M. Then, the mixture solutions were sonicated for 30 min and then kept at least 2 h at room temperature to ensure the formation of micelles. Finally, the fluorescence spectra were collected at the excitation wavelength of 333 nm, an emission wavelength range of 350-435 nm, and slit opening of both excitation and emission at 1.0 mm by QuantMaster-40 fluorescence spectrophotometer (Protein Technologies, Inc., Tucson, AZ). The fluorescence intensity ratio of I_{373} and I_{394} was analyzed as a function of logarithm C11G3-TBP concentration.

Preparation of anionic phosphorus dendron-based nanomedicine C11G3-TBP@Cur. Cur (ranging from 1.54 to 3.85 mg) dissolved in 300 μL MeOH was mixed with C11G3-TBP (10 mg) in 3.0 mL water at different molar ratios (C11G3-TBP: Cur = 1: 10, 1: 15, 1: 20 or 1:25). Each mixture was magnetically stirred at room temperature overnight to leave the volatilization of MeOH. The mixture solution was centrifuged (8000 rpm for 20 min) to remove the precipitates related to non-encapsulated free Cur. The precipitate was collected and dissolved into 1 mL MeOH for UV-vis analysis at 425 nm. The supernatant was lyophilized to obtain the C11G3-TBP@Cur nanomicelles.The drug-loading content and drug encapsulation efficiency were calculated according to the following equations:

$$\text{Drug loading content (\%)} = W_1 / W_2 \times 100\% \quad (1)$$

$$\text{Drug encapsulation efficiency (\%)} = W_1 / W_0 \times 100\% \quad (2)$$

where W_1 represents the loaded amount of Cur within the nanomicelles, W_0 the initial total amount of Cur, and W_2 the amount of drug-loaded nanomicelles.

Cur release from the C11G3-TBP@Cur micelles. To determine the release kinetics of Cur from the C11G3-TBP@Cur micelles, 1 mL of C11G3-TBP@Cur micelle solution in phosphate buffered saline (PBS, pH = 7.4) was placed in a regenerated cellulose dialysis bag (molecular weight cut-off = 5 kDa), which was incubated in 19 mL of PBS containing Tween-80 (0.5% w/w) at 37 °C with gentle shaking. At predetermined time points, 1 mL of the outer phase buffer medium was taken out and 1 mL of the buffer was supplemented to maintain the constant volume of outer phase buffer

medium. The Cur absorption at 425 nm was recorded by UV-vis spectrometry to quantify the concentration of released Cur according to the corresponding absorption-concentration calibration curve.

Transmission electron microscopy (TEM) and atomic force microscope (AFM) imaging. C11G3-TBP and C11G3-TBP@Cur were respectively dissolved into water to reach a final concentration of 2.5 mg/mL, sonicated for 30 min, and then kept at least 2 h at room temperature. After the micelle formation, each sample was diluted to a working concentration of 0.25 mg/mL. Then, a TEM sample was prepared by dropping the aqueous solution of each sample onto a carbon-coated copper grid and air dried before measurements. TEM imaging was executed using a JEOL 2010 analytical electron microscope (JEOL, Tokyo, Japan) at an operating voltage of 200 kV. The particle size distribution was measured using Image J 1.40 G software (http://rsb.info.nih.gov/ij/download.html). For each sample, at least 100 particles from different images were randomly selected and analyzed. Besides, an AFM sample was prepared by dropping an aqueous particle suspension onto a silicon wafer and nitrogen-dried before measurements. AFM imaging was carried out using a Molecular Force Probe 3D analytical electron microscope (Asylum Research, Santa Barbara, CA) to observe the size and morphology of micelles.

Hydrodynamic size and zeta-potential measurements. C11G3-TBP and C11G3-TBP@Cur were respectively dispersed in water (1 mg/mL), sonicated for 5 min, and then kept at least 2 h at room temperature. The nanomicelles were diluted to have a concentration of 0.1 mg/mL, and dynamic light scattering and zeta-potential measurements were carried out using a Malvern Zetasizer Nano-ZS Nanoseries 3 equipped with a standard 633-nm laser (Worcestershire, UK). All measurements were performed at room temperature, and three parallel measurements were performed for each sample.

Fluorescence emission spectra. Free Cur and C11G3-TBP@Cur solutions with the same Cur concentration were separately prepared. Then, the fluorescence emission spectra were recorded by QuantMaster-40 fluorescence spectrometer (Protein Technologies Inc., Tucson, AZ) at the excitation wavelength of 425 nm. The emission slit opening was set at 1.0 mm.

Cytotoxicity assay. The cytotoxicity of C11G3-TBP@Cur, C11G3-TBP or Cur was assessed by

CCK-8 viability assay of activated MH-S cells. Concisely speaking, 8000 cells were seeded into each well of 96-well plates with 100 μL Roswell Park Memorial Institute 1640 (RPMI 1640) medium containing 10% FBS, 0.05 mM β-mercaptoethanol, 100 U/mL penicillin, and 100 U/mL streptomycin at 37 °C and 5% CO_2 overnight. The next day, the cells were incubated with 2 μg/mL lipopolysaccharide (LPS) for 24 h. Then, the medium was replaced with fresh medium containing C11G3-TBP@Cur, C11G3-TBP or Cur (10 μL in PBS) at different Cur or equivalent Cur concentrations and cells treated with PBS were used as control. The cells were incubated at 37 °C for 24 h. Finally, the medium was replaced with fresh RPMI 1640 medium (without FBS) containing CCK-8 (10% v/v), and cells were incubated at 37 °C for 3 h. Then, each well of cells was measured using a Thermo Scientific Multiskan MK3 enzyme linked immunosorbent assay (ELISA) reader (Thermo Scientific, Waltham, MA) at a wavelength of 450 nm and five parallel wells were assayed for each sample.

***In vitro* cellular uptake of C11G3-TBP@Cur nanomicelles.** MH-S cells were seeded in 12-well plates at a density of 1×10^5 cells per well, cultivated overnight, and LPS-activated for 24 h. Then, the macrophages were treated with the free Cur (dissolved in 1% DMSO) or C11G3-TBP@Cur (different formulations containing Cur under the Cur concentration ranging from 2.5 to 10 μM) in serum-containing RPMI 1640 medium for 4 h at 37 °C. After that, the cells were washed with PBS for three times, digested with trypsin-EDTA, resuspended in PBS after centrifugation (1000 rpm, 5 min), and finally assayed by flow cytometry *via* Becton Dickinson Facscan analyzer (Franklin Lakes, NJ) in the FL1-H channel with each sample measured for three times.

To qualitatively evaluate the cellular uptake efficiency, confocal microscopic imaging was performed. Concisely, LPS-activated MH-S cells were seeded in confocal dishes at a density of 1×10^5 cells per dish (2 mL RPMI 1640 medium) at 37 °C overnight. Then, medium of each dish was replaced with fresh medium containing C11G3-TBP@Cur or Cur (different formulations containing Cur under the Cur concentration of 10 μM) for 4 h. Thereafter, cells were washed with PBS and stained with DAPI for 10 min. In the end, the cells were observed by laser scanning confocal microscopy

(ZEISS LSM-700, Jena, Germany) at 63 × oil-immersion objective lens.

Pro-inflammatory cytokine expression *in vitro*. MH-S cells were seeded into 12-well culture plates at a density of 1×10^5 cells per well with 1 mL of fresh RPMI 1640 medium at 37 °C and 5% CO_2 overnight, and activated with 2 μg/mL LPS for 24 h. Then, cells in each well were treated with fresh medium containing free C11G3-TBP, C11G3-TBP@Cur or free Cur (dissolved in 1% DMSO) under different concentrations. Cells treated with PBS were used as control. After cultivation for 24 h, culture medium of each well was collected to analyze the pro-inflammatory factors (TNF-α, IL-1β and IL-6) using commercial ELISA kits, and inflammatory mediator NO using commercial Griess Reagent kits. The cells were collected in centrifuge tubes and washed with PBS for three times. The inflammatory-related mRNA within cells was determined using RT-PCR (model 7500, Applied Biosystems, Waltham, MA) according to the manufacturer's protocols. In brief, total RNA was extracted from the MH-S cells utilizing RNAeasy™ Plus Animal RNA Isolation Kit, and first strand cDNA was generated by BeyoRT™ III First Strand cDNA Synthesis Kit. The expression of pro-inflammatory factors (TNF-α, IL-1β and IL-6), anti-inflammatory factors (IL-10, CD206 and Arg-1) and iNOS was quantified by RT-PCR (n =6). The amplification was performed in 40 cycles by a fluorescence detection system (MJ Research) with SYBR green fluorescence. Each cycle consisted of heat denaturation for 15 s at 95 ℃, annealing for 15 s at 62 ℃, and extension for 30 s at 72 ℃. All samples were quantified using a $2^{-\Delta\Delta Ct}$ method to calculate the relative mRNA expression using the reference gene *β-actin*. The upstream and downstream primer sequences of each gene are shown in Table S1. Meanwhile, after cultivation for 24 h, cells were washed with PBS and collected in 15-mL centrifuge tubes, stained with APC-CD86 and PE-Arg-1 monoclonal antibodies at 0 °C for 30 min in the dark. Flow cytometry was used to analyze the ratio of M1/M2 cell polarization *via* Becton Dickinson Facscan analyzer (Franklin Lakes, NJ). Each sample was tested in triplicate.

NF-κB transcription factor assay *in vitro*. MH-S cells were seeded into 6-well culture plates at a density of 2×10^5 cells per well with 1 mL of fresh RPMI 1640 medium at 37 °C and 5% CO_2 overnight, and activated with 2 μg/mL LPS for 24 h. Then, cells in each well were treated with fresh medium containing drug-free C11G3-TBP, C11G3-TBP@Cur or free Cur (dissolved in 1% DMSO)

at Cur or equivilent Cur concentrtaion of 10 μM and cultivated for 24 h. Cells treated with PBS were used as positive control and normal cells treated with PBS were used as negative control. Extraction of cytoplasmic or nuclear NF-κB proteins from MH-S cells was carried out using commercial Nuclear and Cytoplasmic Protein Extraction Kit. Briefly, cells were collected in centrifuge tubes, washed with PBS for two times, and added with 200 μL lysis buffer containing phenylmethanesulfonyl fluoride under an ice-bath for 30 min. To extract cytoplasmic proteins, MH-S cells were first treated with 200 μL cytoplasmic protein extraction buffer A under vortexing for 5 s. Then, the mixture was added with 10 μL cytoplasmic protein extraction buffer B through 5 s vortexing. After maintained under ice bath for 30 min, the mixture was centrifuged (12000 g, 5 min at 4 ℃) to isolate the supernatant, which was used to extract the cytoplasmic proteins for NF-κB detection. To extract nuclear proteins, the cell pellets were resuspended with 50 μL nuclear protein extraction buffer under vortexing for 15-30 s, maintained under ice bath for 5 min and then vortexed for 20 s every 2 min for a total of 30 min. Finally, the mixture was centrifuged (12000 g, 10 min at 4 ℃) to isolate the nuclear proteins. The obtained nuclear and cytoplasmic proteins were used to detect NF-κB through Western blotting according to the literature protocols.[3] β-Actin and histone were employed as reference proteins, respectively.

Oxidative stress assay. MH-S cells were seeded in 12-well plates at a density of 1×10^5 cells per well (1 mL of RPMI-1640 medium), cultivated to reach 80% confluence, and LPS-activated for 24 h. After being treated with free Cur (dissolved in 1% DMSO) or C11G3-TBP@Cur ([Cur] = 5-20 μM) for 6 h, the cells were incubated with ROS detection agent (ROS Brite™ 670) according to the manufacturer's instruction. Flow cytometric analysis was performed to quantitatively investigate the ROS-positive fluorescence intensity of cells. The cells were rinsed with PBS for three times, digested with trypsin-EDTA, resuspended in 200 μL PBS, and analyzed in the FL2-H channel. Each measurement was sampled with 1×10^4 cells (n = 3). Then, the cells were also fixed with 2.5% glutaraldehyde for 15 min at room temperature, washed with PBS for three times, stained with DAPI for 10 min, and observed by laser scanning confocal microscopy. To further reveal the antioxidative

property of C11G3-TBP@Cur, the antioxidation-related mRNAs (HO-1, SOD-2 and NOX-2) were examined using RT-PCR under the protocols mentioned above. The upstream and downstream primer sequences of each gene are shown in Table S1.

***In vivo* ALI therapy**. All animal experiments were performed in accordance with the guidelines of the Committee on Experimental Animal Care and Use of Donghua University and also following the regulations of the National Ministry of Health. Six-week-old Male Balb/c mice (Shanghai Slac Laboratory Animal Center, Shanghai, China) were intraperitoneally injected with LPS solution (1 mg/mL, in 100 μL PBS for each mouse) to induce ALI. At 24 h post LPS challenge, free Cur (5 mg/kg, 100 μL PBS (1% DMSO)), C11G3-TBP (6.48 mg/kg, 100 μL PBS), and C11G3-TBP@Cur (21.83 mg/kg, 100 μL PBS) were respectively aerosolized and inhaled by each mouse. ALI mice and normal mice treated with PBS were used as positive and negative control, respectively. After 24 h, animals were sacrificed to obtain lung tissues and BALF. Lung tissues were collected and weighed to obtain the "wet" weight, dried at 80 °C for 72 h to obtain the "dry" weight, and the wet/dry weight ratio was calculated to evaluate the anti-inflammatory effect of lung tissue. BALF was centrifuged (1000 rpm for 5 min) to collect the supernatant for quantification of pro-inflammatory and anti-inflammatory factors (TNF-α, IL-1β, IL-6, Arg-1, CD206 and IL-10) and neutrophils infiltration marker MPO using commercial ELISA kits. In addition, the parallel lung tissues were homogenized to collect total RNA *via* a Trizol reagent for RT-PCR determination of the mRNA expression levels of pro-inflammatory and anti-inflammatory factors (TNF-α, IL-1β, IL-6, Arg-1, CD206 and IL-10). The assay was carried out according to the aforementioned protocols and *GAPDH* was employed as a reference gene. Further, the homogenized lung tissues were treated with Nuclear and Cytoplasmic Protein Extraction Kit to determine the nuclear and cytoplasmic NF-κB protein content through Western blotting [3].

***In vivo* biodistribution.** To investigate the biodistribution of the C11G3-TBP@Cur nanomicelles, each ALI mouse was administrated with the nanomicelles (21.83 mg/kg, 100 μL PBS) according to the above protocols. At different time points (12, 24, 48 and 96 h) post-administration, each mouse was euthanized. The heart, liver, spleen, lung and kidney were extracted, treated with *aqua regia* for 5 days, and diluted by water to 4 mL for each sample. The Cur concentration of each sample (n = 3)

was measured by QuantMaster-40 fluorescence spectrometer (Protein Technologies Inc., Tucson, AZ) at the excitation wavelength of 425 nm.

Histological examinations and micro-computed tomography (micro-CT) imaging. One mouse of each group was euthanized at 24 h post treatment. Then, the heart, liver, spleen, lung and kidney were harvested and sectioned for hematoxylin-eosin (H&E) staining, while the lung tissue was also sectioned for ROS detection probe staining. Both stainings were carried out according to standard protocols [4]. Each mouse was scored according to the severity of inflammatory lung injury following four items: alveolar congestion, haemorrhage, infiltration or aggregation of neutrophils in the vessel wall, and thickness of the alveolar wall, where 0 = minimal (little) damage, 1 = mild damage, 2 = moderate damage, 3 = severe damage, and 4 = maximal damage [5]. Fluorescence probe staining was performed to detect ROS content in lung tissue with the DHE probe (1: 400 diluted), and visualized by Nikon DS-U3 Detection System (Tokyo, Japan) according to standard protocols [6]. Meanwhile, another mouse of each group was euthanized and lung tissues were harvested and fixed in paraformaldehyde (4%) at room temperature for 48 h. The mouse lung tissues were also assessed by micro-CT system (SCANCO Medical AG, Zurich, Switzerland). CT scanning parameters were set at a voltage of 45 kV, a current of 200 μA, and an exposure time of 300 ms. The volumes of lung tissue were measured from reconstructed three dimensional CT images.

Statistical analysis. A one-way analysis of variance statistical method was adopted to evaluate the significance of the data for comparison of in-between groups using IBM SPSS Statistic 26 software (IBM, Armonk, NY). A value of 0.05 was considered as the level of significance, and the associated data were indicated as (*) for $p < 0.05$, (**) for $p < 0.01$, (***) for $p < 0.001$, respectively.

Table S1. The sequences of upstream, downstream, and stem loop primers.

Factors	Sense	Antisense
TNF-α	5′- AGTGGAGGAGCAGCTGGAGT -3	5′-TCCCAGCATCTTGTGTTTCTG-3′
IL-1β	5′-CAACCAACAAGTGATATTCTCCATG-3′	5′-ATCCACACTCTCCAGCTGCA-3′
IL-6	5′-TTCTTGGGACTGATGCTG-3′	5′-CTGGCTTTGTCTTTCTTGTT-3′
iNOS	5’-TCCTGGAGGAAGTGGGCCGAAG-3’	5’ -CCTCCACGGGCCCGGTACTC-3’
HO-1	5′-AGGTACACATCCAAGCCGAGA-3′	5′-CATCACCAGCTTAAAGCCTTCT-3′
SOD-2	5′-CAGACCTGCCTTACGACTATGG-3′	5′-CTCGGTGGCGTTGAGATTGTT-3′
NOX-2	5′- GACAGGAACCTCACTTTCCATA -3′	5′- TGAAGAGATGTGCAATTGTGTG -3′
Arg-1	5′-GAGAAGGTCTCTACATCACAGAAG-3′	5′-TTCACAGTACGAGTCACCTCC-3′
CD206	5′-GTTTCCATCGAGACTGCTGC-3′	5′-GCCACTTTCCTTCAACATTCG-3′
IL-10	5′-GCTCTTACTGACTGGCATGAGGA-3′	5′-AGGAGTCGGTTAGCAGTATGTTG-3′
β-actin	5′-ACGGTCAGGTCATCACTATCG-3′	5′-GTTTCATGGATGCCACAGGATT-3′
NADPH	5′-AAGGGTCATCATCTCTGCCC-3′	5′-GTGATGGCATGGACTGTGGT-3′

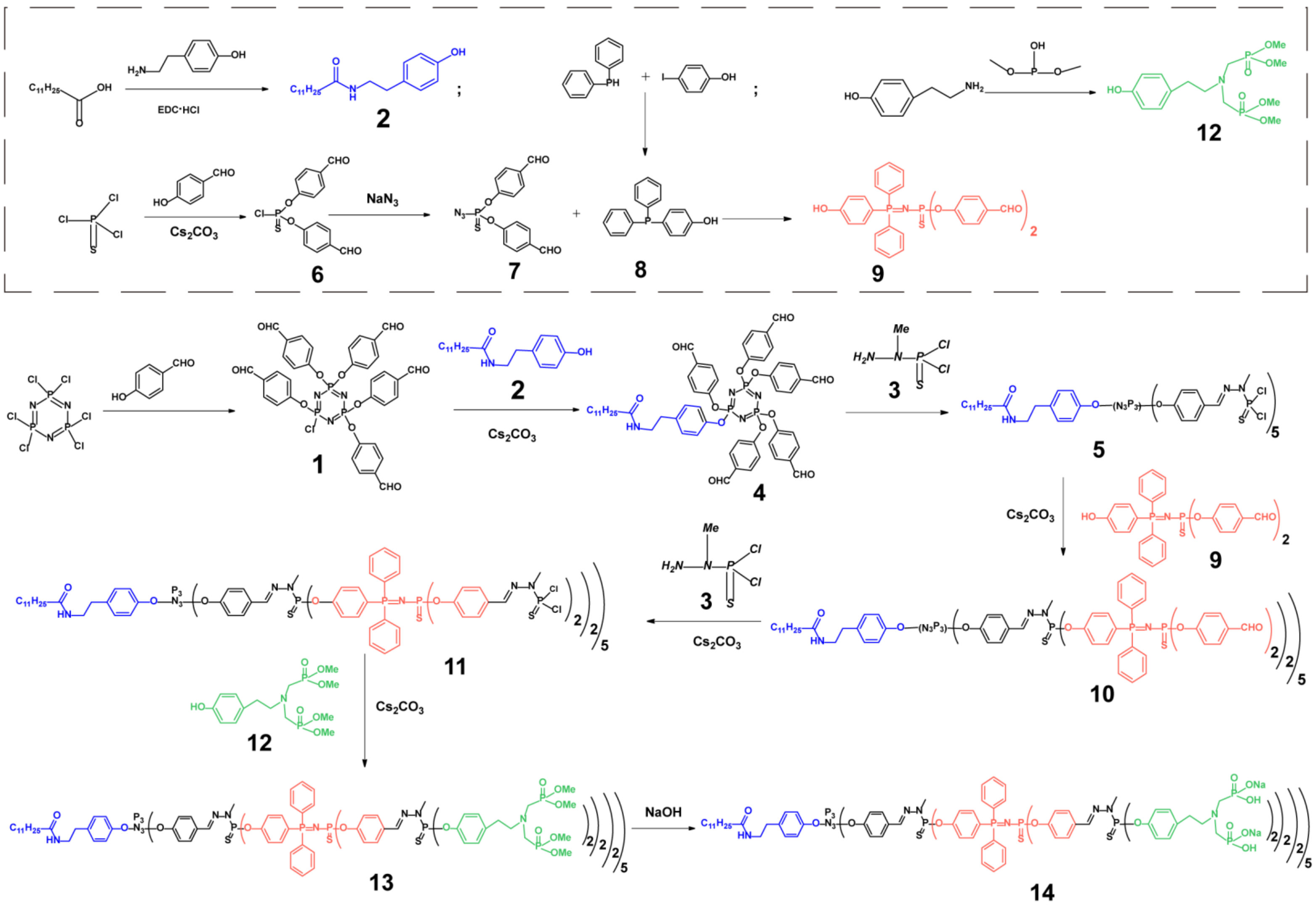

Figure S1. Synthesis of anionic phosphorous dendrons (C11G3-TBP).

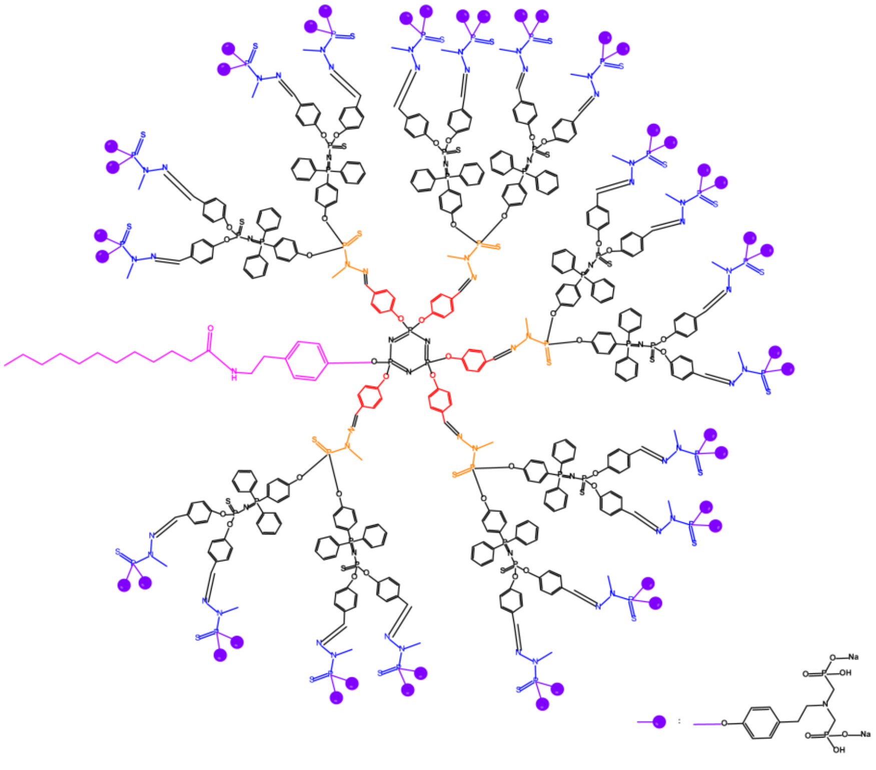

Figure S2. 2D chemical structures of C11G3-TBP dendron.

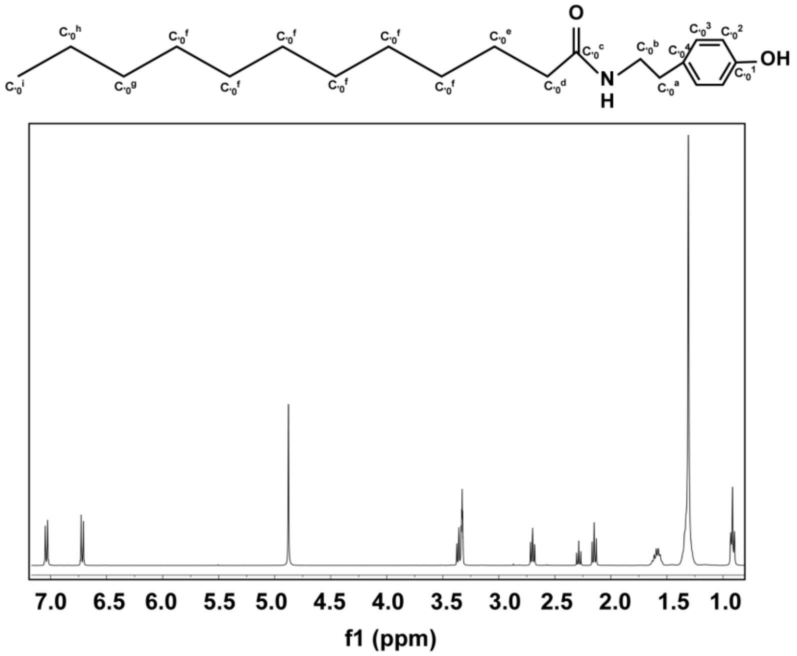

Figure S3. ^{1}H NMR spetrum of compound 2.

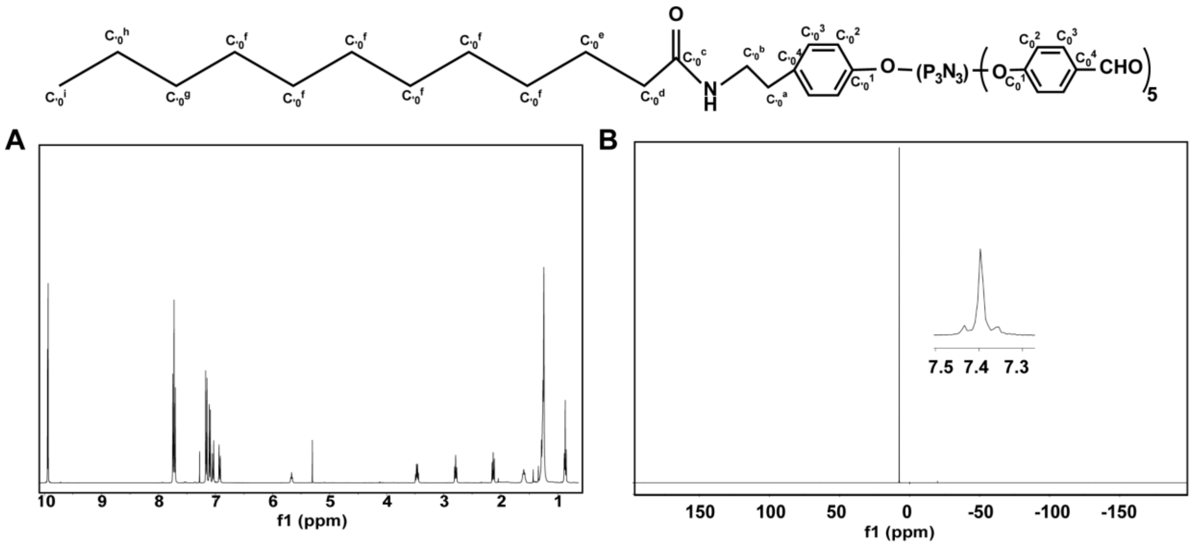

Figure S4. ^{1}H NMR (A) and $^{31}P\{^{1}H\}$ NMR (B) spectra of compound 4.

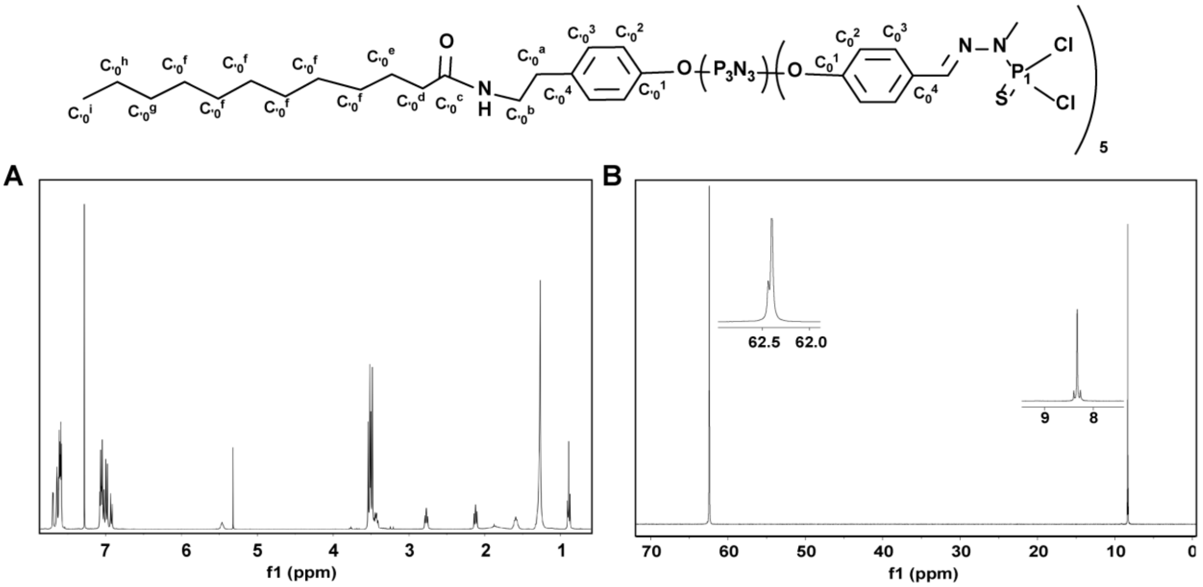

Figure S5. ^{1}H NMR (A) and $^{31}P\{^{1}H\}$ NMR (B) spectra of compound 5.

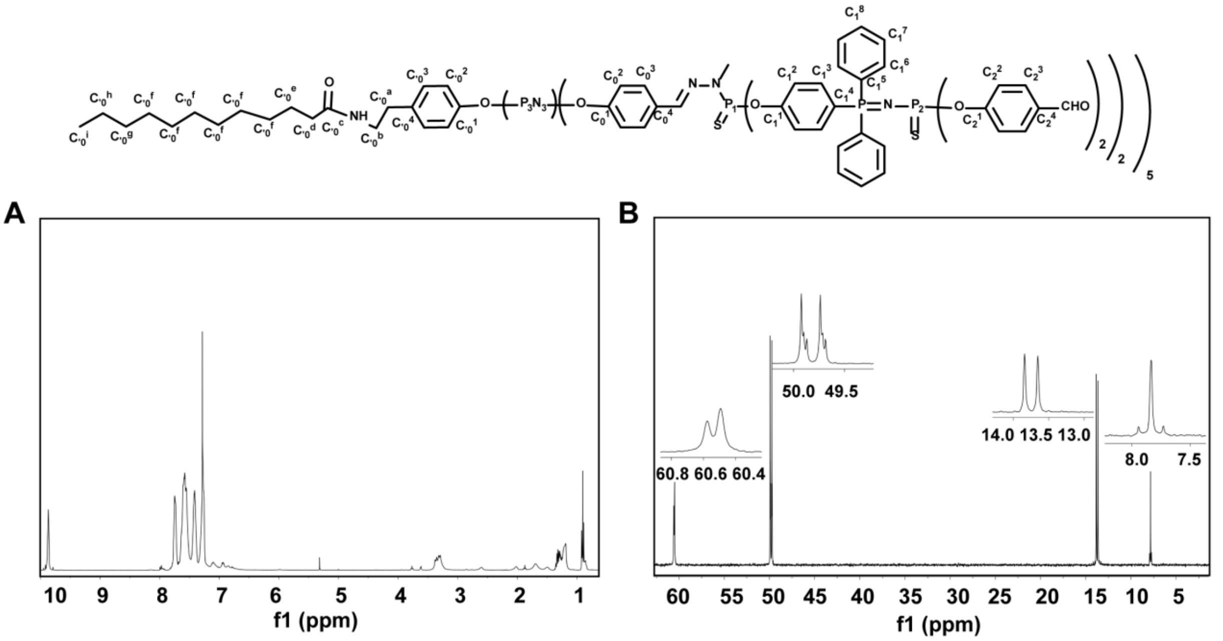

Figure S6. ^{1}H NMR (A) and $^{31}P\{^{1}H\}$ NMR (B) spectra of compound 10.

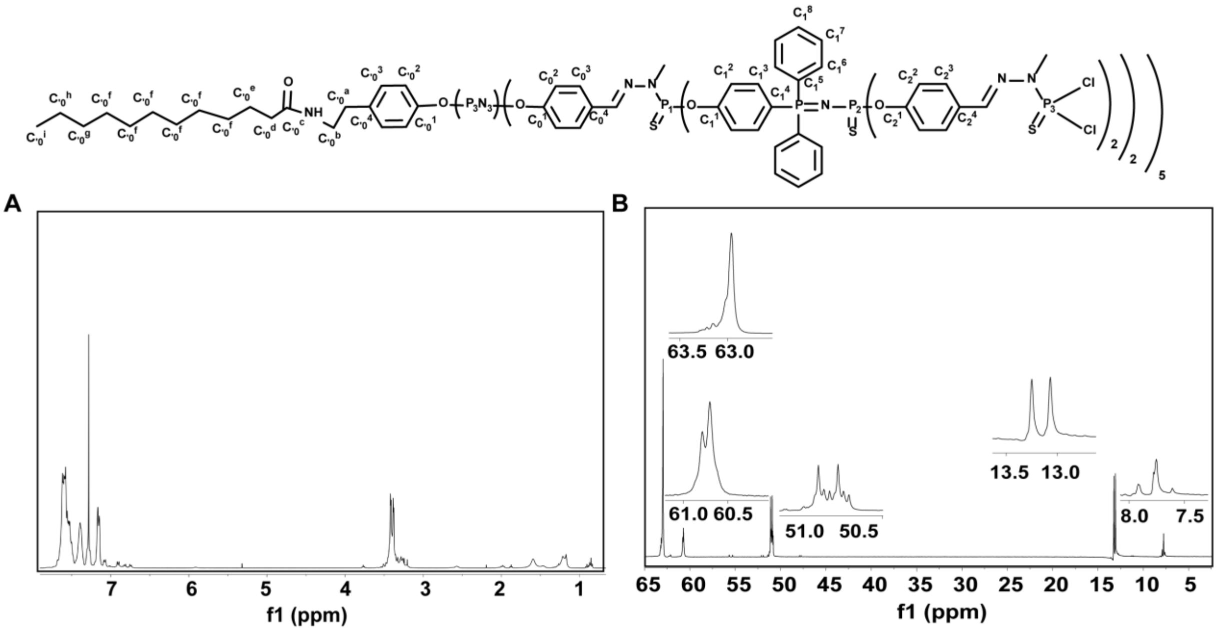

Figure S7. ^{1}H NMR (A) and $^{31}P\{^{1}H\}$ NMR (B) spectra of compound 11.

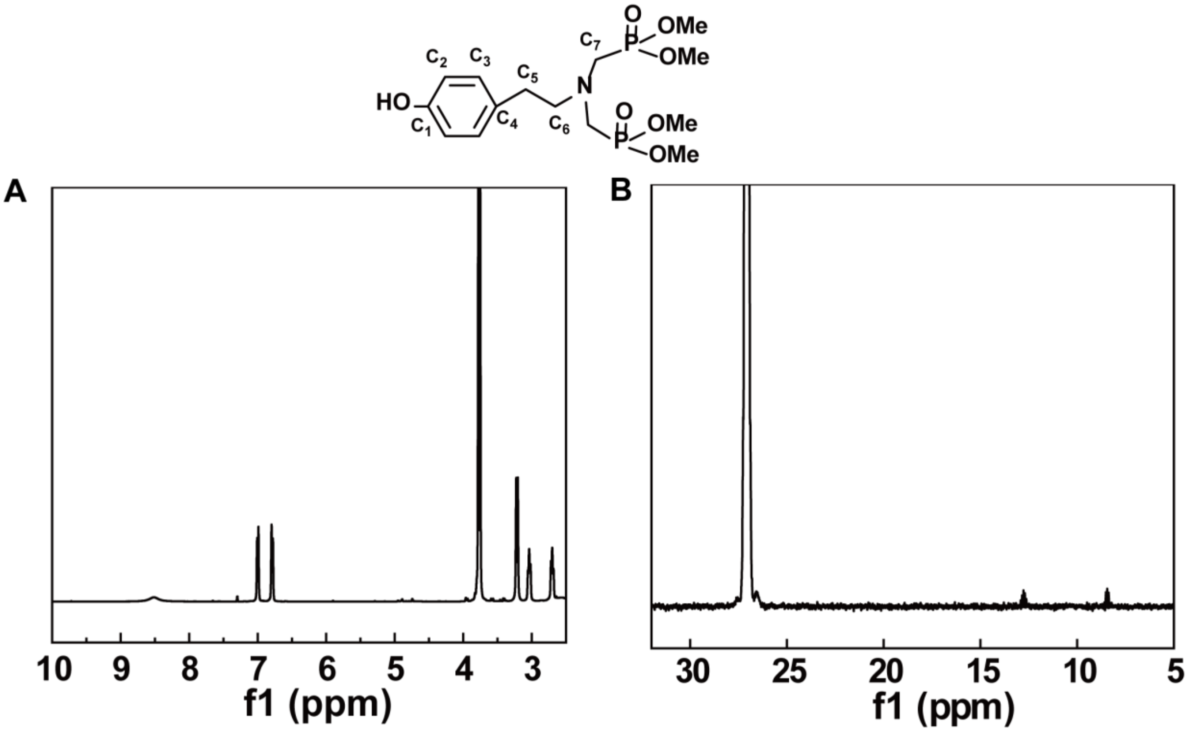

Figure S8. ^{1}H NMR (A) and $^{31}P\{^{1}H\}$ NMR (B) spectra of compound 12.

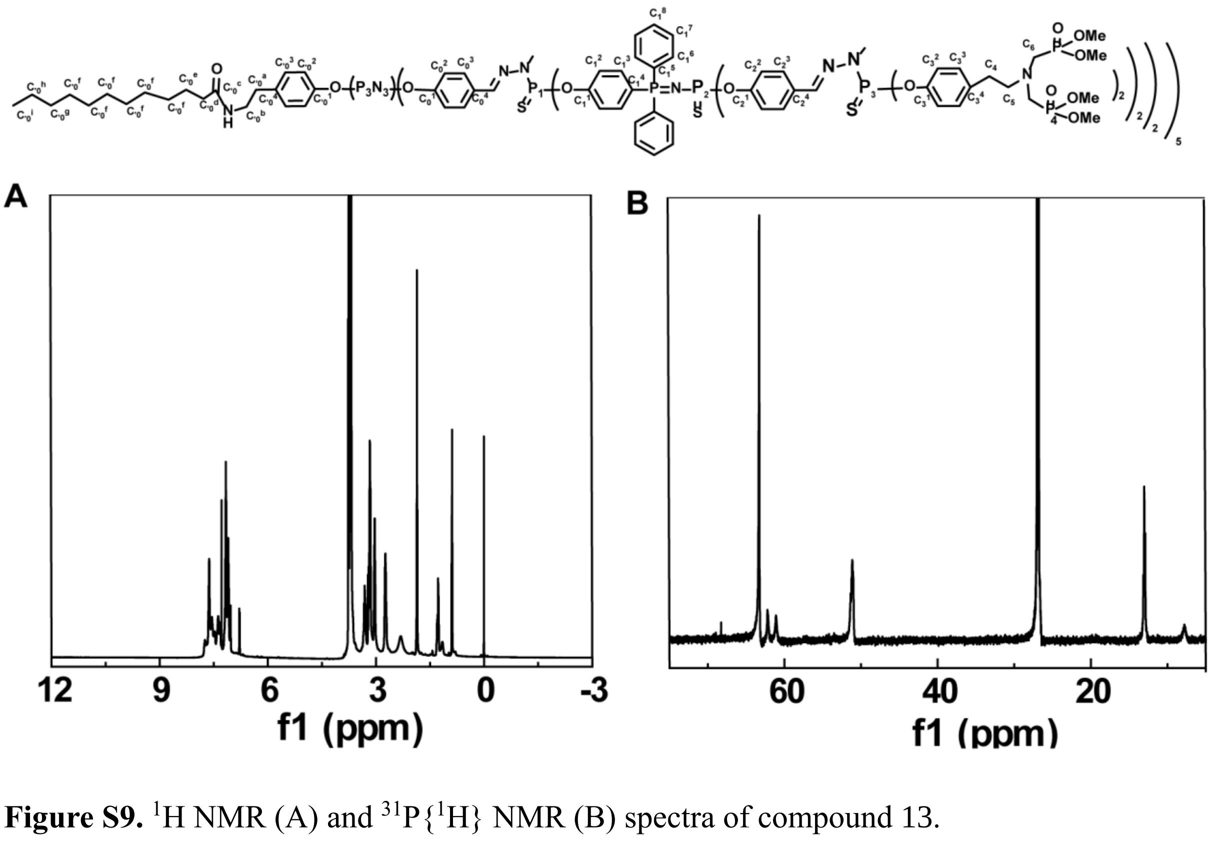

Figure S9. ^{1}H NMR (A) and $^{31}P\{^{1}H\}$ NMR (B) spectra of compound 13.

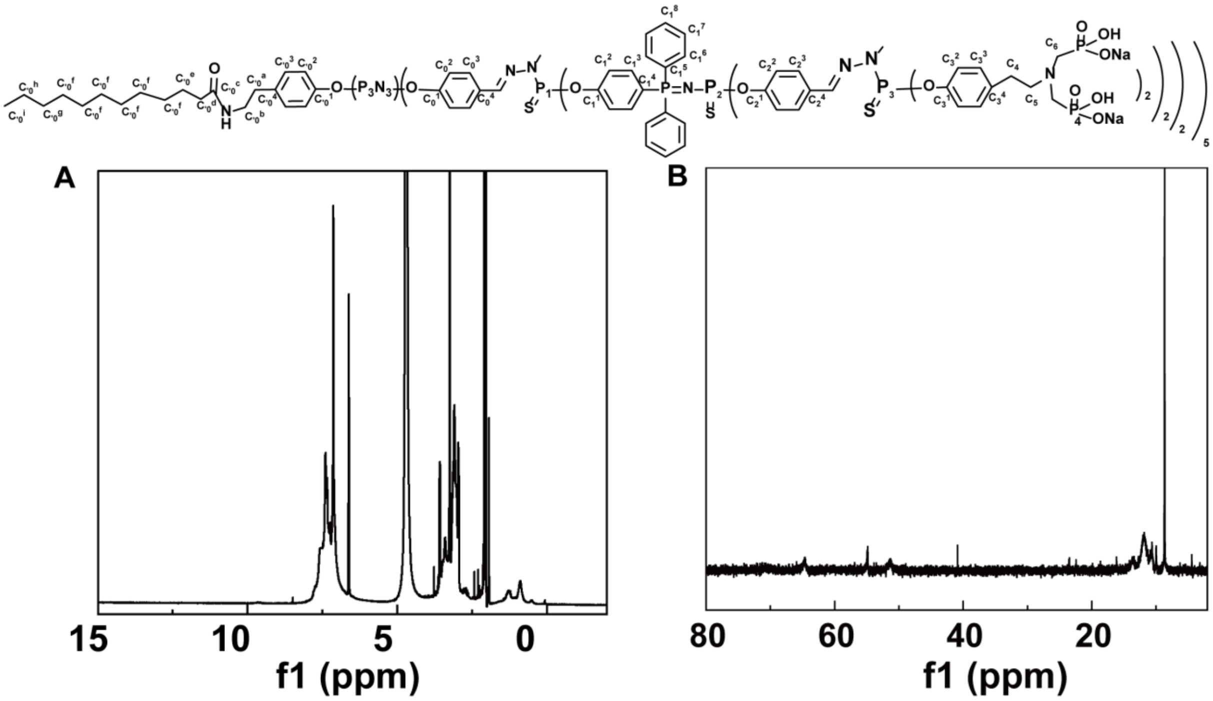

Figure S10. ^{1}H NMR (A) and $^{31}P\{^{1}H\}$ NMR (B) spectra of compound 14.

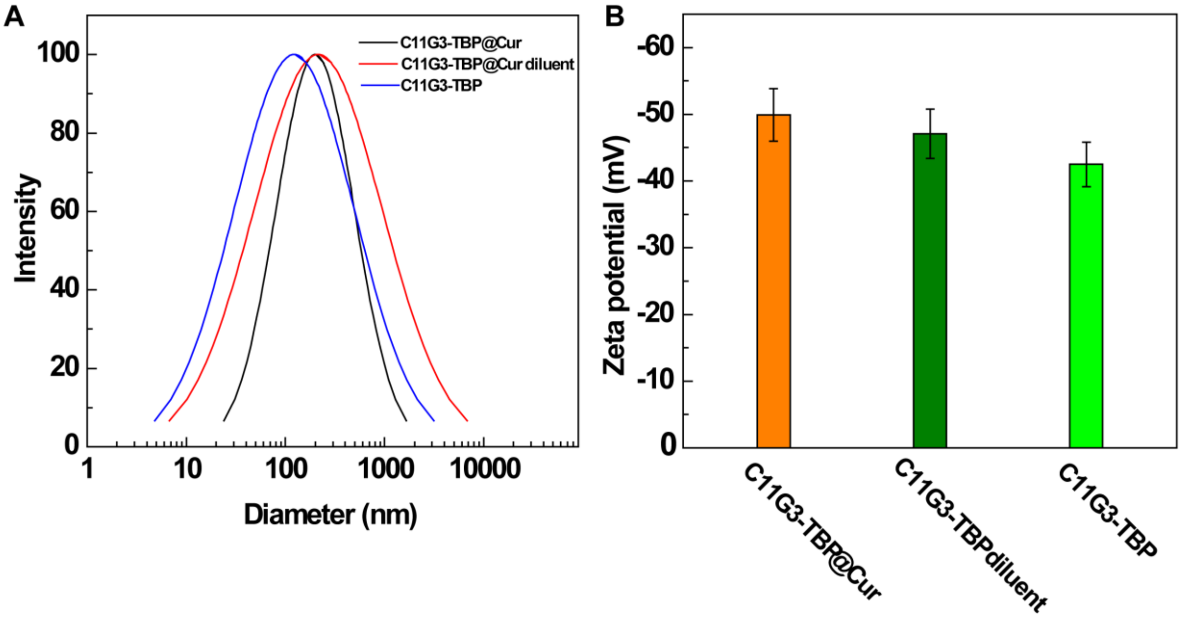

Figure S11. Dynamic light scattering (A) and zeta potential (B) analysis of C11G3-TBP nanomicelles (41.7 μM), C11G3-TBP@Cur nanomicelles ([C11G3-TBP] = 41.7 μM) and C11G3-TBP@Cur nanomicelle diluent ([C11G3-TBP] = 4.17 μM). The zeta-potential data are shown as mean ± SD (n = 3).

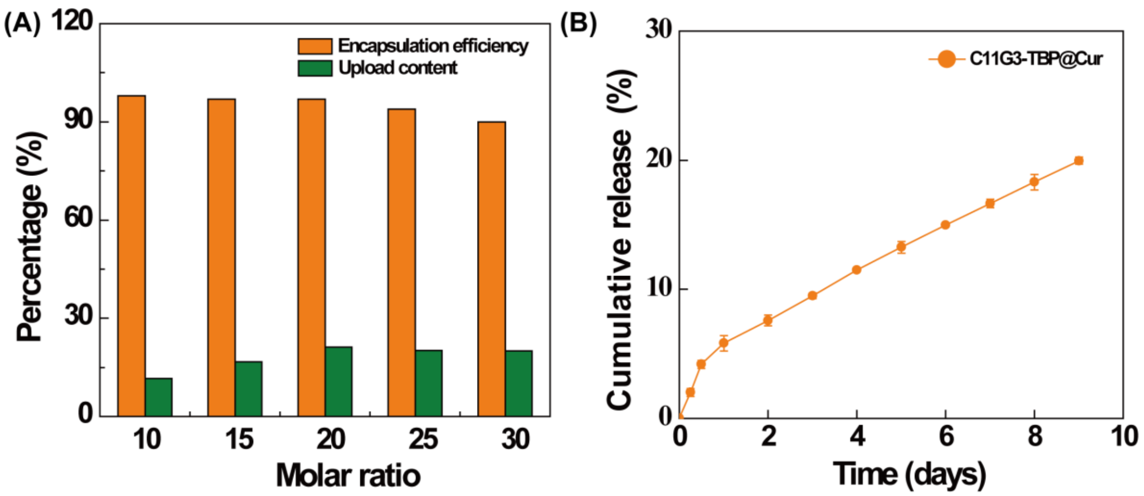

Figure S12. (A) Screening the ratios of C11G3-TBP nanomicelles and the antioxidative drug Cur for the optimal drug-loading content and drug-encapsulating efficiency. (B) *In vitro* release profile of C11G3-TBP@Cur micelles in PBS solution (pH = 7.4) at 37 ℃ (n = 3).

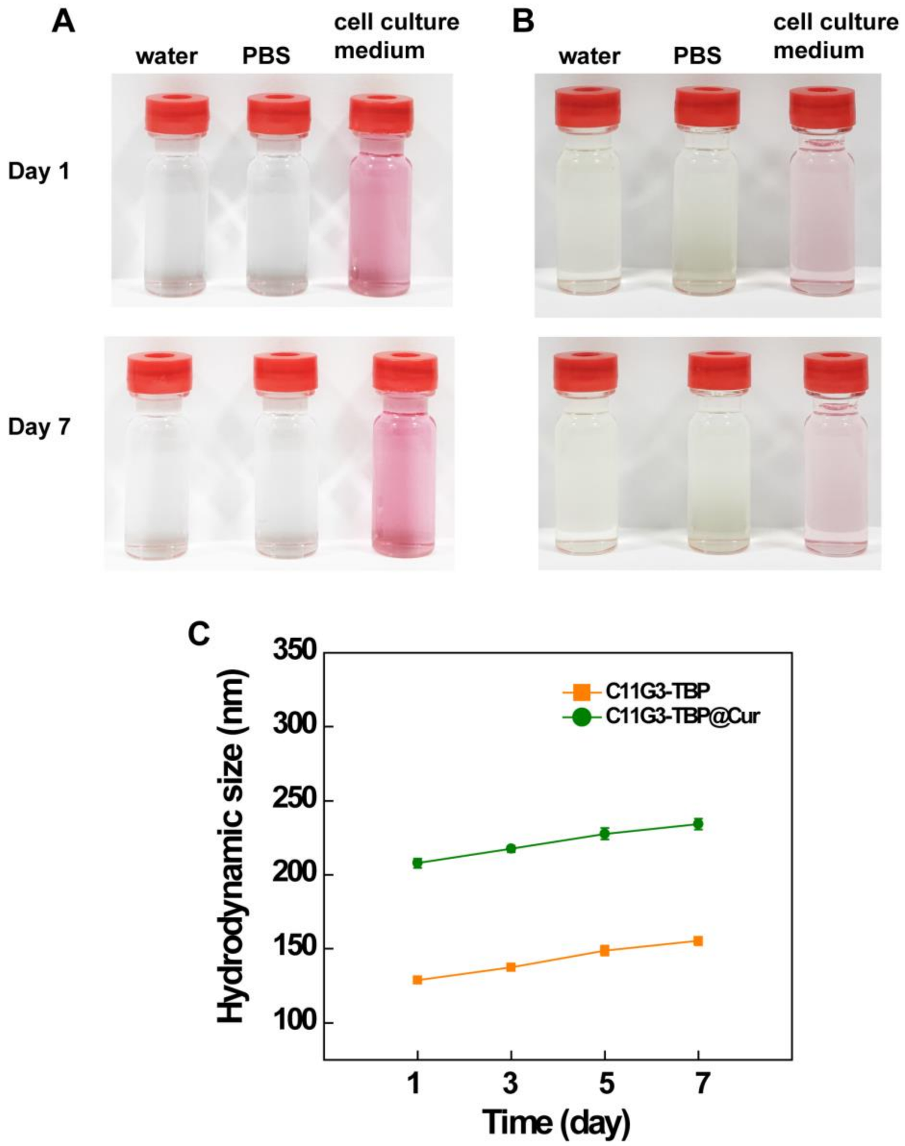

Figure S13. Photos of the aqueous solutions of C11G3-TBP (A) and C11G3-TBP@Cur (B) nanomicelles dispersed in different aqueous solvents at a relative C11G3-TBP concentration of 41.7 μM. (C) Time-dependent hydrodynamic size change of C11G3-TBP and C11G3-TBP@Cur nanomicelles dispersed in water (n = 3).

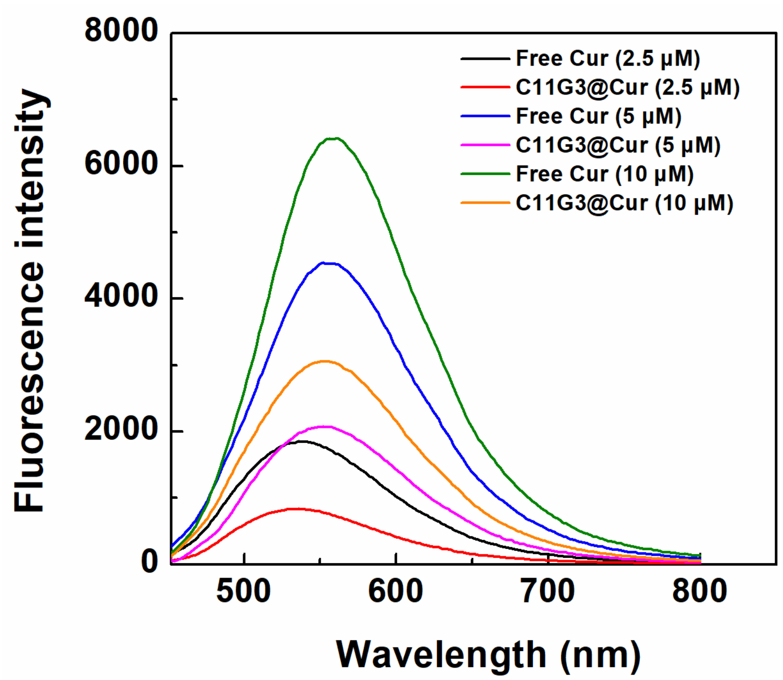

Figure S14. Fluorescence emission spectra of C11G3-TBP@Cur nanomicelles and free Cur (the equivalent concentration of Cur was from 2.5 to 10 μM for both materials).

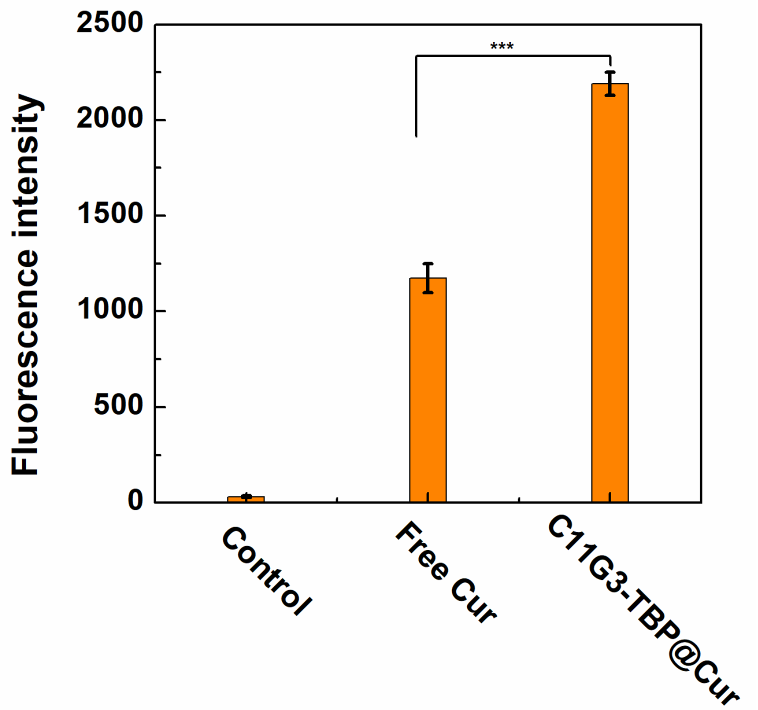

Figure S15. The quantitative analysis of Cur fluorescence in LPS induced MH-S cells after 4 h incubation with different materials at the Cur or equivalent Cur concentration of 10 μM (n = 3).

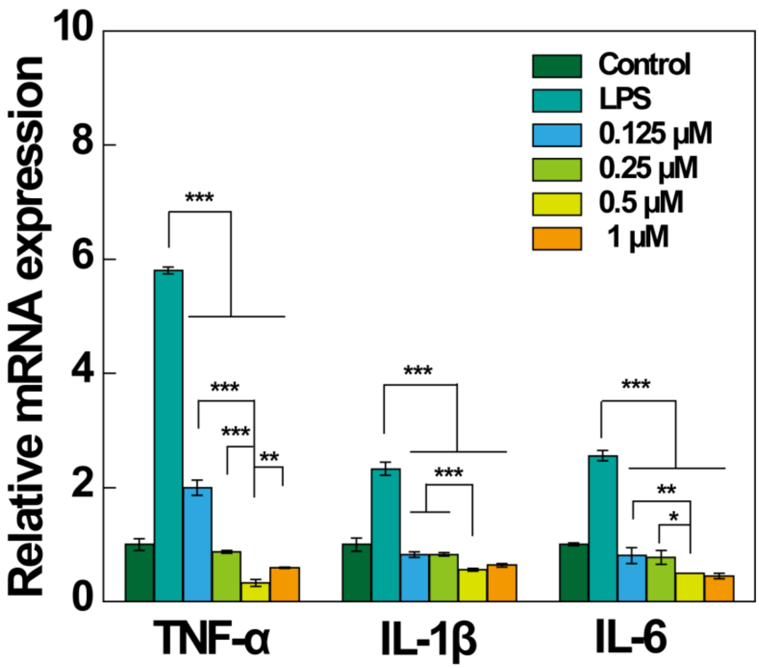

Figure S16. RT-PCR assay of mRNA expressions of the pro-inflammatory cytokines (TNF-α, IL-1β and IL-6) in LPS-activated MH-S cells after treatment with C11G3-TBP nanomicelles under different concentrations (n = 3).

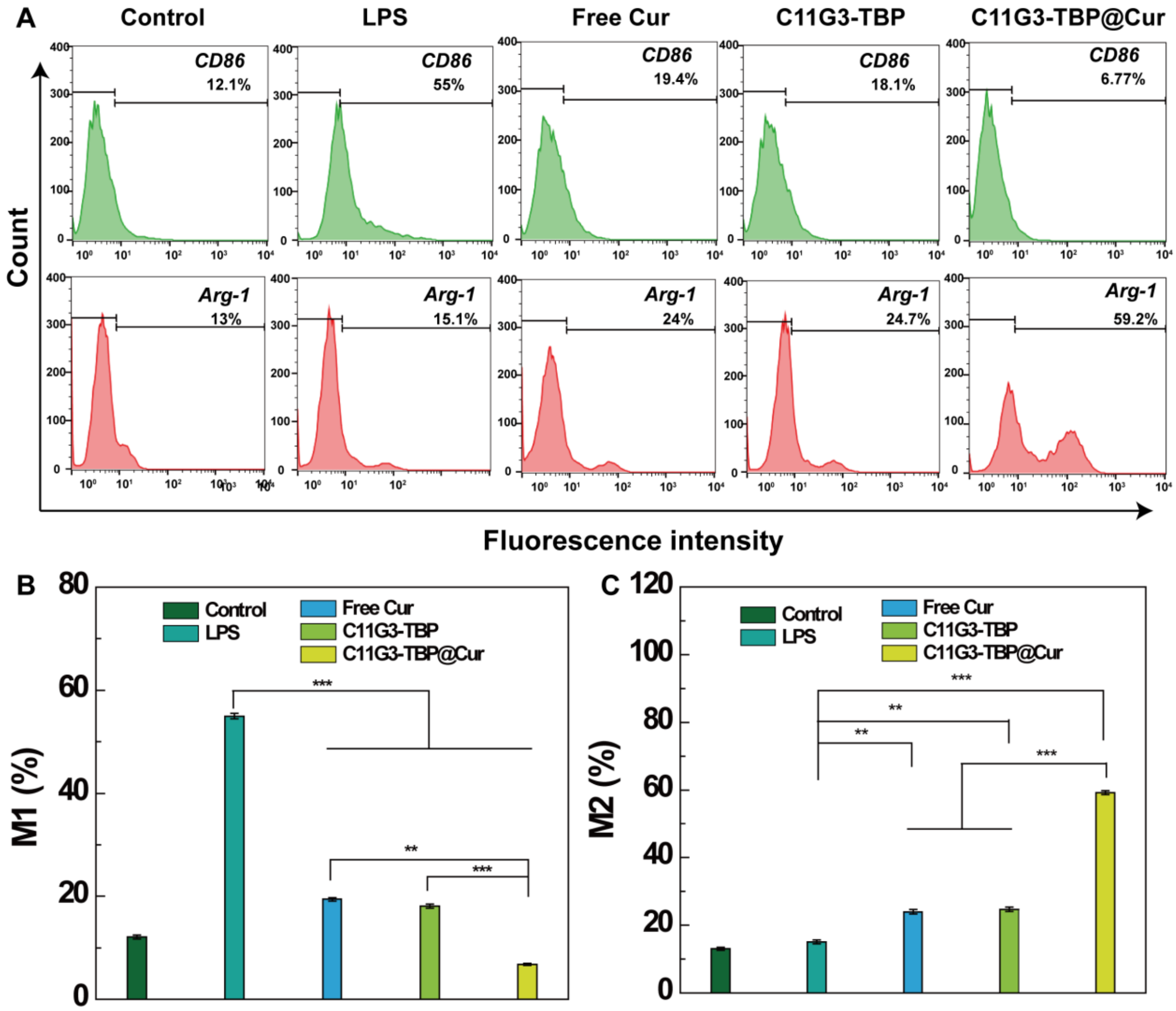

Figure S17. (A) The expression ratio of CD86 and Arg-1 treated with free Cur, C11G3-TBP nanomicelles and C11G3-TBP @Cur nanomicelles in MH-S cells *via* flow cytometry assay. (B) and (C) The quantitative analysis of M1 and M2 percentage in different treatment groups (n = 3).

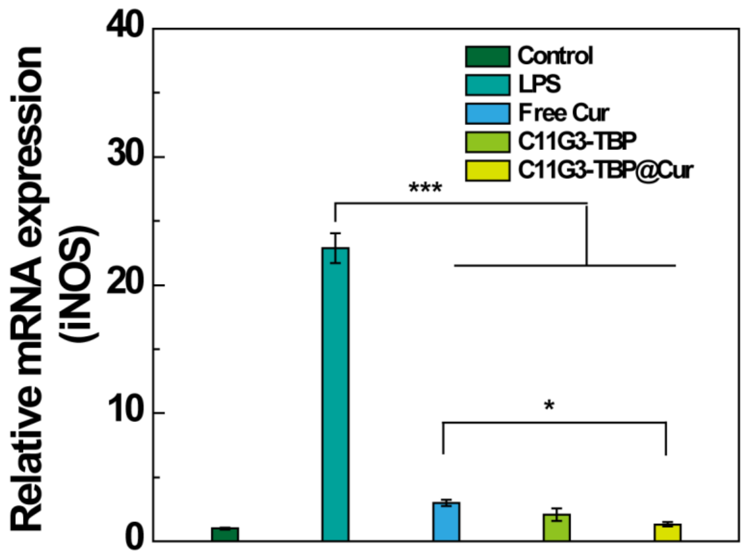

Figure S18. RT-PCR assay of mRNA expression of iNOS after the LPS-activated MH-S cells were treated with different materials at the Cur or equivalent Cur concentration of 10 μM (n = 3).

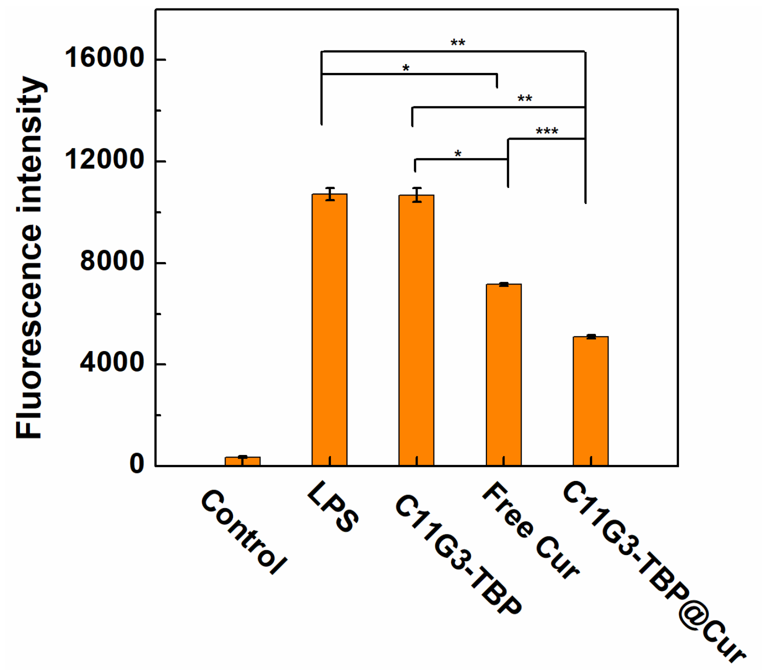

Figure S19. The quantitative analysis of ROS Brite™ 670 probe fluorescence in LPS-induced MH-S cells after 6 h incubation with different materials at the Cur or equivalent Cur concentration of 10 μM (n = 3).

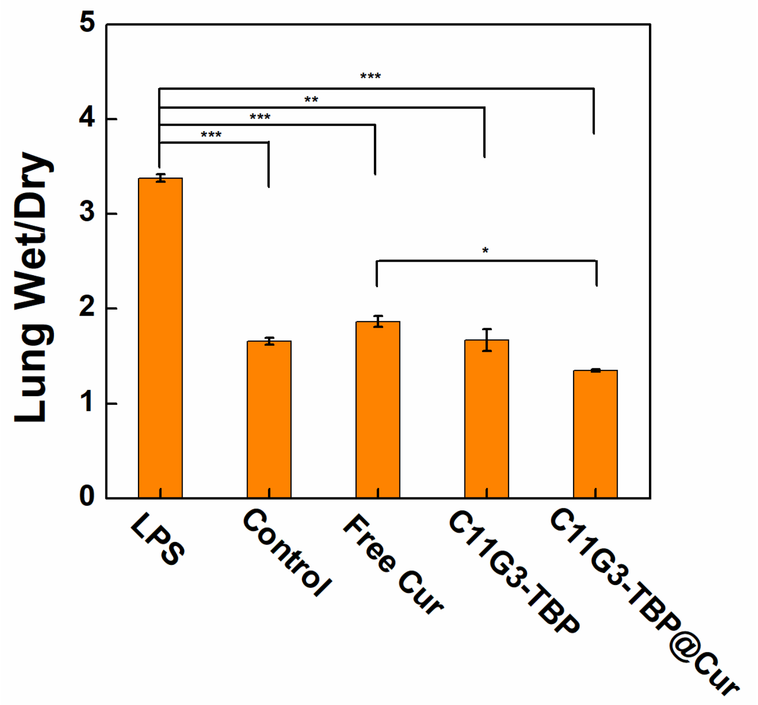

Figure S20. The lung tissue wet/dry weight ratios at 24 h post treatment in different groups (n = 3).

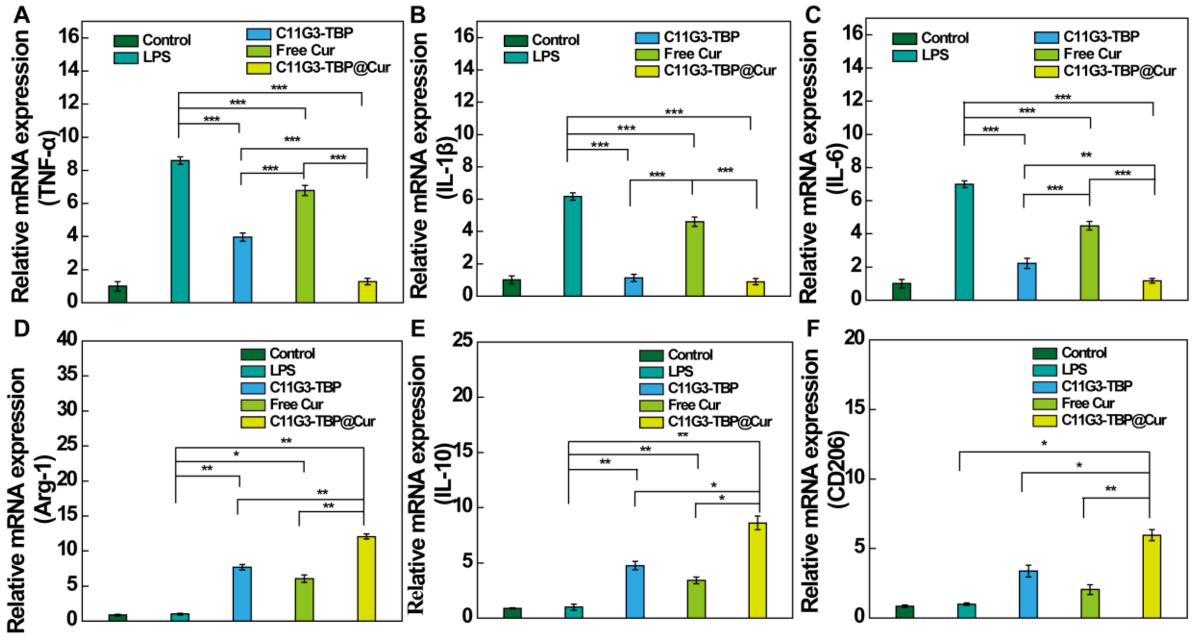

Figure S21. RT-PCR assay of mRNA levels of pro-inflammatory cytokines of (A) TNF-α, (B) IL-1β and (C) IL-6, and anti-inflammatory cytokines of (D) Arg-1, (E) IL-10 and (F) CD206, respectively in injured lung tissues after different treatments (n = 3).

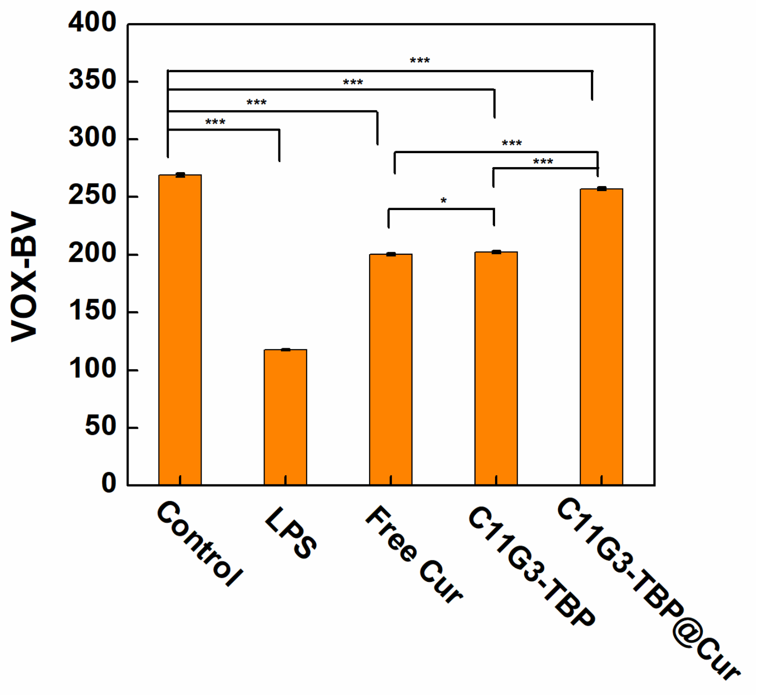

Figure S22. The lung tissue volumes (VOX-BV) of mice at 24 h post treatment in different groups (n = 3).

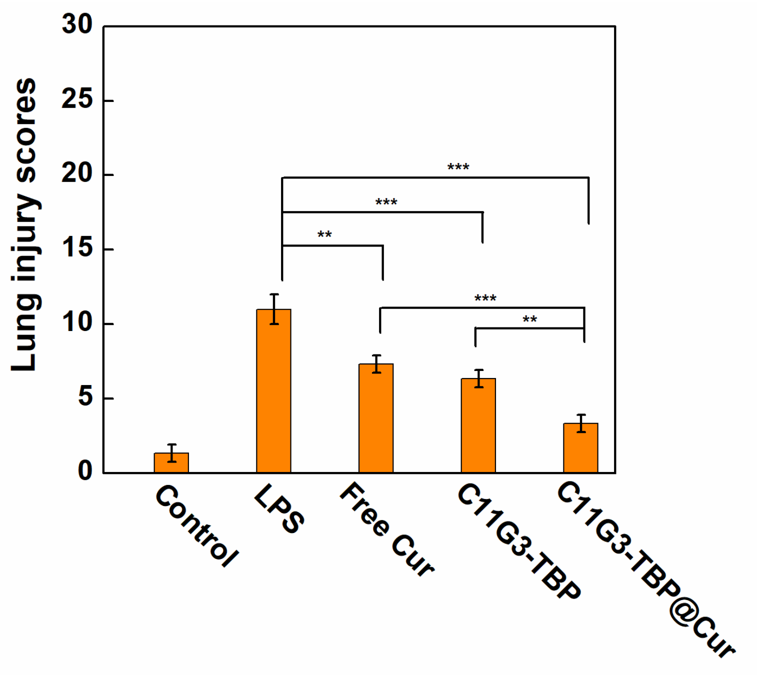

Figure S23. The lung injury scores of mice at 24 h post treatment in different groups (n = 3).

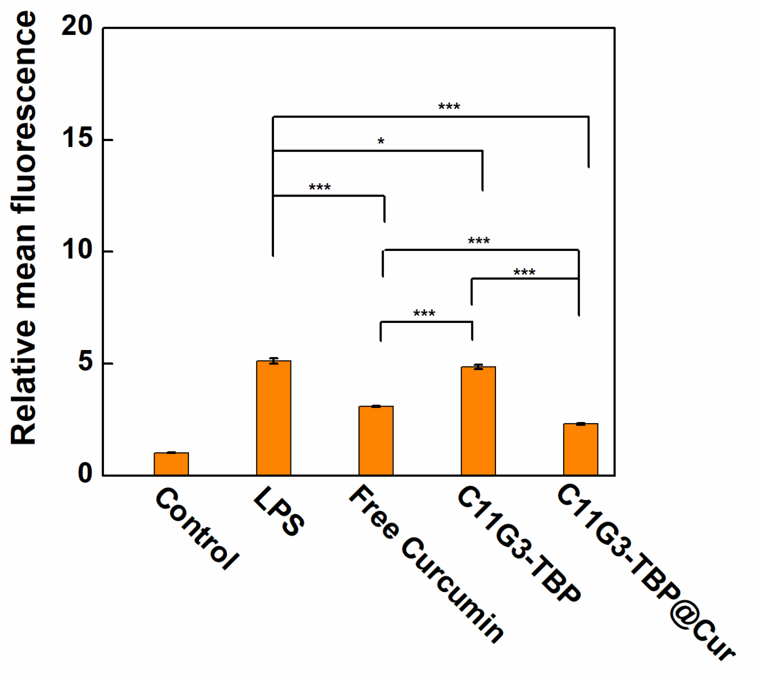

Figure S24. The quantitative analysis of DHE fluorescence in lung sections at 24 h post treatment in different groups (n = 3).

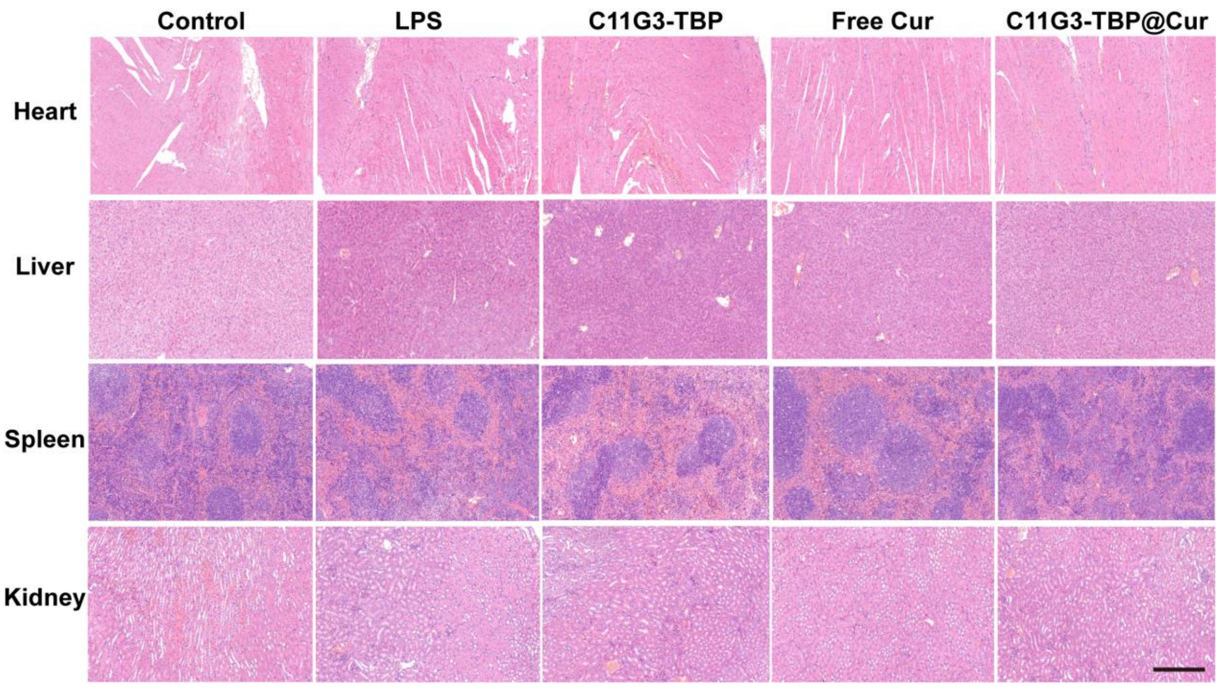

Figure S25. H&E-stained images of major organs (heart, liver, spleen, and kidney) extracted from different groups at 24 h post treatment. Normal mice administrated with PBS were used as negative control and LPS-induced ALI mice administrated with PBS were used as positive control. Scale bar in the panel represents 200 μm.

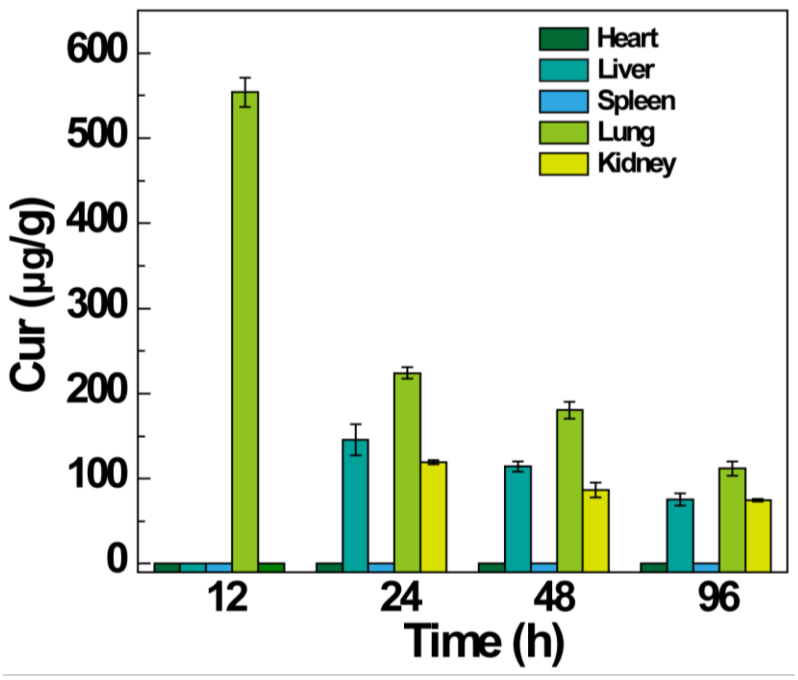

Figure S26. Biodistribution of Cur in major organs of ALI mice at 24 h, 48 h and 96 h post-administration of C11G3-TBP@Cur nanomicelles (the equivalent concentration of Cur was 5 mg/kg). All data are shown as mean ± SD (n = 3).

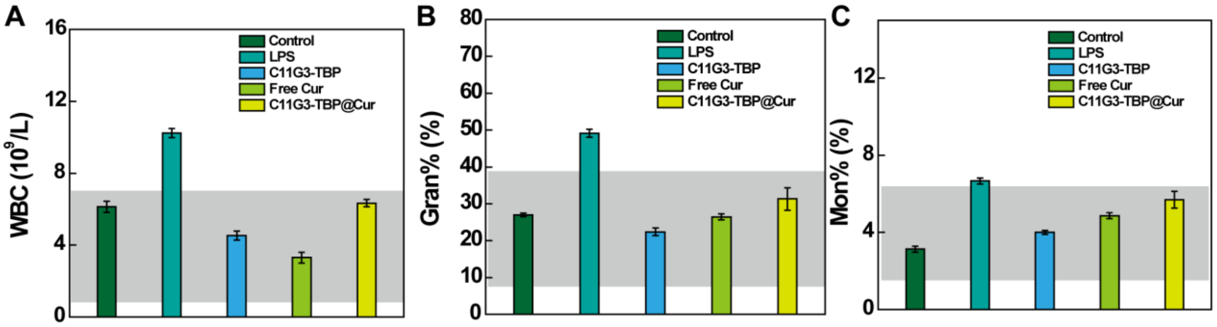

Figure S27. ALI mice after intratracheal atomization administration by C11G3-TBP nanomicelles, free Cur or C11G3-TBP@Cur nanomicelles for 24 h were sacrificed for blood collection. The blood routines involved blood levels of (A) white blood cells (WBC), (B) percentage of neutrophil granulocyte (Gran%) and (C) percentage of monocytes (Mon%). Gray areas indicate the referred normal ranges for healthy mice obtained from Servicebio, Inc. (Wuhan, China) using Hematology Analyzer (Mindray, BC-2800 vet). The data are shown as mean ± SD (n = 3). As opposed to the LPS positive control, all treatment groups show the normal range of the respective indicators.

References

1. Chen L, Fan Y, Qiu J, Laurent R, Li J, Bignon J, *et al*. Potent anticancer efficacy of first-in-class Cu-II and Au-III metaled phosphorus dendrons with distinct cell death pathways. Chemistry. 2020; 26: 5903-10.

2. Katir N, El Brahmi N, El Kadib A, Mignani S, Caminade A, Bousmina M, *et al*. Synthesis of onion-peel nanodendritic structures with sequential functional phosphorus diversity. Chemistry. 2015; 21: 6400-8.

3. Kong L, Wu Y, Alves C, Shi X. Efficient delivery of therapeutic siRNA into glioblastoma cells using multifunctional dendrimer-entrapped gold nanoparticles. Nanomedicine (Lond). 2016; 11: 3103-15.

4. Zhu J, Zheng L, Wen S, Tang Y, Shen M, Zhang G, *et al*. Targeted cancer theranostics using alpha-tocopheryl succinate-conjugated multifunctional dendrimer-entrapped gold nanoparticles. Biomaterials. 2014; 35: 7635-46.

5. Zhao T, Zhao HW, Li G, Zheng SF, Liu MJ, Gu CP, *et al*. Role of the PKC alpha-c-Src tyrosine kinase pathway in the mediation of p120-catenin degradation in ventilator-induced lung injury. Respirology. 2016; 21: 1404-10.

6. Wang L, Cheng X, Li H, Qiu F, Yang N, Wang B, *et al*. Quercetin reduces oxidative stress and inhibits activation of c-Jun N-terminal kinase/activator protein-1 signaling in an experimental mouse model of abdominal aortic aneurysm. Mol Med Rep. 2014; 9: 435-42.
